# G-quadruplex in the TMV Genome Regulates Viral Proliferation and Acts as Antiviral Target of Photodynamic Therapy

**DOI:** 10.1371/journal.ppat.1011796

**Published:** 2023-12-07

**Authors:** Congbao Xie, Xianpeng Zhang, Wenyue Pei, Ju Sun, Hongqi Shang, Zhiyuan Huang, Mengxi Wang, Daozhong Wang, Guiqian Wang, Zhikun Gui, Sisi Liu, Feng Li, Dengguo Wei

**Affiliations:** 1 National Key Laboratory of Agricultural Microbiology, College of Veterinary Medicine, Huazhong Agricultural University, Wuhan, Hubei, China; 2 Hubei Hongshan Laboratory, Interdisciplinary Sciences Institute, Huazhong Agricultural University, Wuhan, Hubei, China; 3 National Reference Laboratory of Veterinary Drug Residues (HZAU) and National Safety Laboratory of Veterinary Drug (HZAU), MOA Key Laboratory for Detection of Veterinary Drug Residues, MOA Laboratory for Risk Assessment of Quality and Safety of Livestock and Poultry Products, Wuhan, Hubei, China; 4 Shenzhen Institute of Nutrition and Health, Huazhong Agricultural University, Wuhan, Hubei, China; 5 Shenzhen Branch, Guangdong Laboratory for Lingnan Modern Agriculture, Genome Analysis Laboratory of the Ministry of Agriculture, Agricultural Genomics Institute at Shenzhen, Chinese Academy of Agricultural Sciences, Shenzhen, China; 6 Frontiers Science Center for Animal Breeding and Sustainable Production, Wuhan, Hubei, China; 7 College of Plant Science and Technology, Huazhong Agricultural University, Wuhan, Hubei, China; 8 National Key Laboratory for Germplasm Innovation and Utilization of Horticultural Crops, College of Horticulture and Forestry Sciences, Huazhong Agricultural University, Wuhan, Hubei, China; 9 College of Informatics, Huazhong Agricultural University, Wuhan, Hubei, China; 10 College of Chemistry, Huazhong Agricultural University, Wuhan, Hubei, China; The Ohio State University, UNITED STATES

## Abstract

Plant viruses seriously disrupt crop growth and development, and classic protein-targeted antiviral drugs could not provide complete protection against them. It is urgent to develop antiviral compounds with novel targets. Photodynamic therapy shows potential in controlling agricultural pests, but nonselective damage from reactive oxygen species (ROS) unexpectedly affects healthy tissues. A G-quadruplex (G4)-forming sequence in the tobacco mosaic virus (TMV) genome was identified to interfere the RNA replication *in vitro*, and affect the proliferation of TMV in tobacco. *N*-methyl mesoporphyrin IX stabilizing the G4 structure exhibited inhibition against viral proliferation, which was comparable to the inhibition effect of ribavirin. This indicated that G4 could work as an antiviral target. The large conjugate planes shared by G4 ligands and photosensitizers (PSs) remind us that the PSs could work as antiviral agents by targeting G4 in the genome of TMV. Chlorin e6 (Ce6) was identified to stabilize the G4 structure in the dark and selectively cleave the G4 sequence by producing ROS upon LED-light irradiation, leading to 92.2% inhibition against TMV *in vivo*, which is higher than that of commercial ningnanmycin. The inhibition of Ce6 was lost against the mutant variants lacking the G4-forming sequence. These findings indicated that the G-quadruplex in the TMV genome worked as an important structural element regulating viral proliferation, and could act as the antiviral target of photodynamic therapy.

## Author summary

Plant viruses cause significant losses in crops worldwide, and there is an urgent need to develop antiviral compounds with novel targets. Drugs targeting RNA have attracted much attention, but RNA structures as the antiviral targets remain to be explored. In this study, a photosensitizer, chlorin e6, was identified to cleave a G4 forming sequence in the TMV genome by producing ROS upon LED-light irradiation and to exhibit inhibition against TMV higher than that of commercial Ningnanmycin. This is the first time to show that the folding of G4 regulates the proliferation of plant viruses, which illustrates the viral replication mechanism from the RNA structural perspective. Compounds stabilizing G4 structures or breaking G4 forming sequences are expected to be the novel antivirals against the plant viruses.

## 1 Introduction

Annual crop losses of more than 20% are caused by plant viruses, and chemical compounds are employed to prevent and control these plant viruses. However, classic protein-targeting antiviral drugs cannot effectively inhibit plant viruses *in vivo* [1]. Photodynamic therapy (PDT) exhibits great potential in controlling fungi, bacteria, and insects in agricultural fields [2]. In PDT, photosensitizers (PSs) are activated upon light irradiation to generate reactive oxygen species (ROS) that directly damage the biological tissues or microbial components without causing the development of resistance [3]. However, nonselective damage by ROS can result in unexpected damage to healthy tissues [4]. Specifically targeting functional elements in plant viruses is the key to the development of PSs.

Guanine (G)-rich sequences may form a non-canonical nucleic acid structure called G-quadruplex (G4), which plays an important role in cellular processes such as replication, transcription, and translation of the viral RNA genomes [5]. Small molecule binders of G4 have recently garnered increasing attention due to their high antiviral efficacy [6]. However, many studies regarding G4 are limited to animal viruses, and the study on the G4 in the plant viruses has only recently begun [7]. Exploring G4 function in plant virus genomes sheds light on the prevention and control of plant virus-related diseases. Generally, G4 ligands have large conjugate planes, similar to PS structures [8], which prompted us to design G4-targeting PSs against plant virus genomes.

In this study, we focused on the tobacco mosaic virus (TMV), known for its agricultural threat, as it affects over 885 plant species across 65 families. This virus inflicts damage on crops like tobacco, potato, tomato, among others, contributing to a significant decline in crop yield [9]. We identified a putative G4-forming sequence (PQS) at the coding region of the TMV genome that interfered with RNA synthesis *in vitro* and affected TMV proliferation in *Nicotiana benthamiana* (*N*. *benthamiana*). A classic G4 ligand, N-methyl mesoporphyrin IX (NMM), and some plant secondary metabolites interact with G4 in the TMV genome to produce antiviral effects. Our results suggested that G4 is an important regulator between viruses and plants and that G4 binding compounds in plants affect its folding or unfolding to regulate biological processes, and to achieve harmony between viruses and hosts.

When a PS binds to the G4 in the viral genome, ROS produced upon photoirradiation would specifically disrupt G4 at the local site, thereby inhibiting viral proliferation [10]. In this study, we screened for the G4 binding compounds from a PS library (Chart 1). The PS chlorin e6 (Ce6) was identified to bind with TMV G4 *in vitro*. Upon photoirradiation, Ce6 specifically cleaved G4, rather than ssRNA, by producing ROS, and exhibited stronger anti-TMV activity than commercial ningnanmycin. G4 is a novel target for antiviral compounds; thus, designing small molecules to regulate their stability or breaking the nucleic acid sequences by photocleavage could be an effective approach to controlling plant viruses.

## 2. Results

### 2.1 Identification of G4-forming sequences in TMV genome

A total of 1789 reference genome sequences of plant viruses from 35 classified families (S1 Table) were collected from the National Center for Biotechnology Information (https://www.ncbi.nlm.nih.gov/). All putative G4 sequences (PQSs) on both strands were predicted by quadpaser [11]. The detailed PQS information in viral genomes were uploaded to database Viral Putative G-quadruplex Database (ViPGD) (http://jsjds.hzau.edu.cn/MBPC/ViPGD/index.php/home/index) to facilitate users to visually browse or search PQSs in different viruses [12].

**Fig 1:**
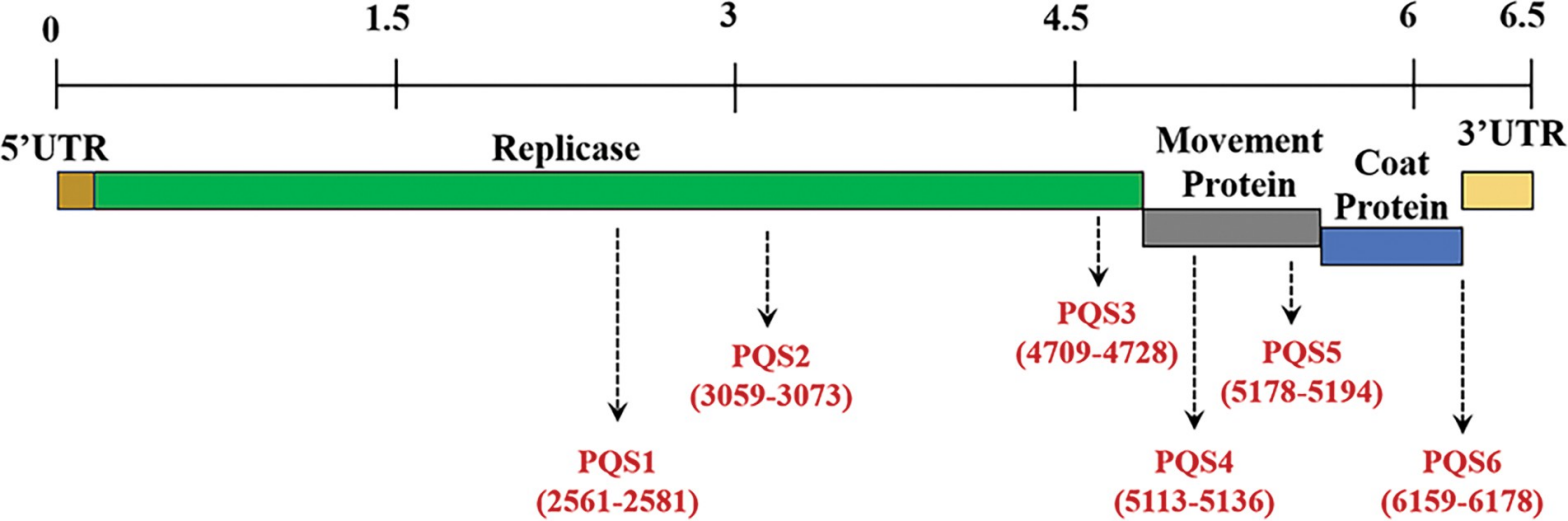
Distribution and pattern conservation of G-PQSs in the genome of TMV. The length of the dash arrows represents the relative pattern conservation of the PQSs in the positive strand.

TMV, a model plant virus, has been widely used in virology study and antiviral agent discovery [13]. A total of six PQSs were identified in the TMV genome (NC_001367.1), of which PQS1, PQS2, and PQS3 were located in the coding region of the replicase; PQS4 and PQS5 were in the coding region of the movement protein, and PQS6 was located in the coding region of the coat protein (Fig 1). Pattern conservation, sequence conservation, and folding potential of PQSs were determined to evaluate the functional potential of PQSs in the genome (Fig 1 and S2 Table) [12].

**Fig 2:**
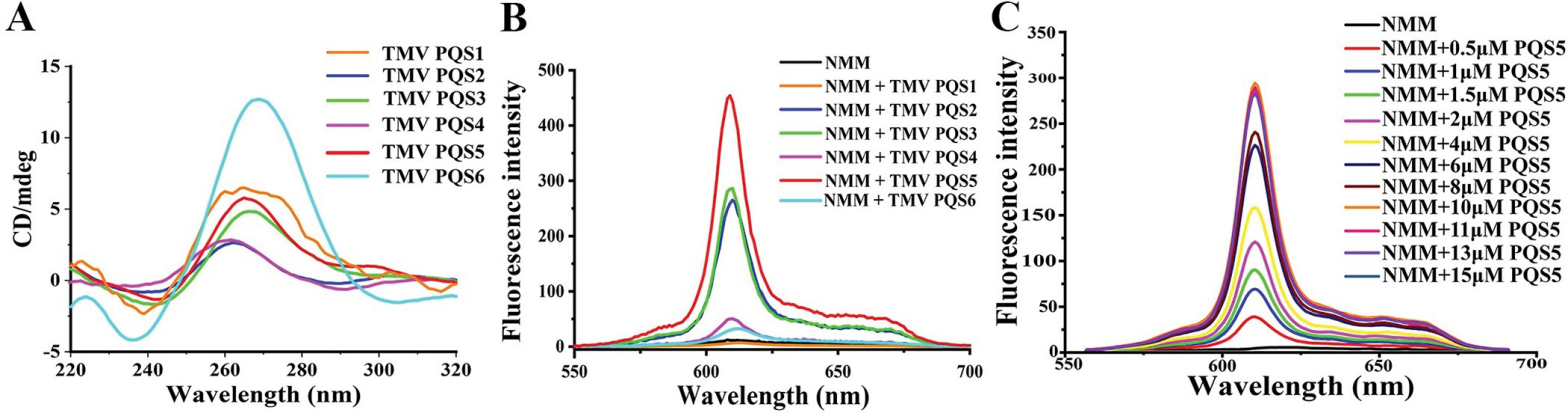
PQS5 exhibits typical G4 characteristics in the solution. (A) CD spectra of PQSs (15 μM) in the genome of TMV. (B) Fluorescence response of NMM to different PQSs. The concentration of NMM and RNA were 5 μM. (C) Fluorescence response of NMM (1.5 μM) to PQS5 with increasing concentrations (0–15 μM), λ_ex_ = 400 nm.

Different conformations of G4 exhibit their characteristic signals in circular dichroism (CD) spectra [14]. The CD spectroscopy of TMV PQS1 suggested that this sequence could fold into multiple conformations in solution (Fig 2A). All CD spectra of TMV PQS2, PQS3, PQS4, and PQS5 exhibited a negative peak at 240 nm and a positive peak at 263–265 nm, which indicated that these PQSs could fold into parallel G4s. The PQS6 showed the strongest CD signals; however, the negative peak at 235 nm and the notable positive peak at 270 nm deviated from the CD spectra of G4s with typical conformations [8]. The existence of G4 conformation in solutions requires further experimental validation.

NMM does not show fluorescence in solution by itself, but it exhibits strong fluorescence upon binding to G4s, thereby functioning as a light-up probe of G4 [15]. The fluorescence signal was the strongest when NMM was mixed with TMV PQS5, moderate when mixed with PQS2 or PQS3, extremely low with PQS4 or PQS6. No fluorescence signal was observed when NMM was mixed with PQS1 (Fig 2B). This indicated that TMV PQS5 (5’-GGUGGACAAAAGGAUGG-3’) can fold into a more stable G4 structure compared with the other TMV PQSs at room temperatures and in solution with 100 mM potassium. Imino proton peaks at 10.5–12.0 ppm in the ^1^H nuclear magnetic resonance spectroscopy indicated G4 structure formation in the solution (S1 Fig). Titration of TMV PQS5 oligonucleotides into the solution with NMM increased the fluorescence emission intensity of NMM at 611 nm in a dose-dependent manner. From the titration process, the dissociation constant (*Kd*) between NMM and G4 could be derived as 3.58 μM (Fig 2C). Single-molecule fluorescence resonance energy transfer (sm FRET) experiment demonstrated that TMV PQS5 could fold into a specific structure, and high concentrations of potassium ions and NMM benefited the folding of this structure (S2 and S3 Figs). Based on the analysis from smFRET, NMR, and CD spectra, we conclude that TMV PQS5 is able to fold into a monomolecular G-quadruplex under near-physiological conditions.

### 2.2 G4 Formation by PQS5 interferes with RNA synthesis *in vitro*

**Fig 3:**
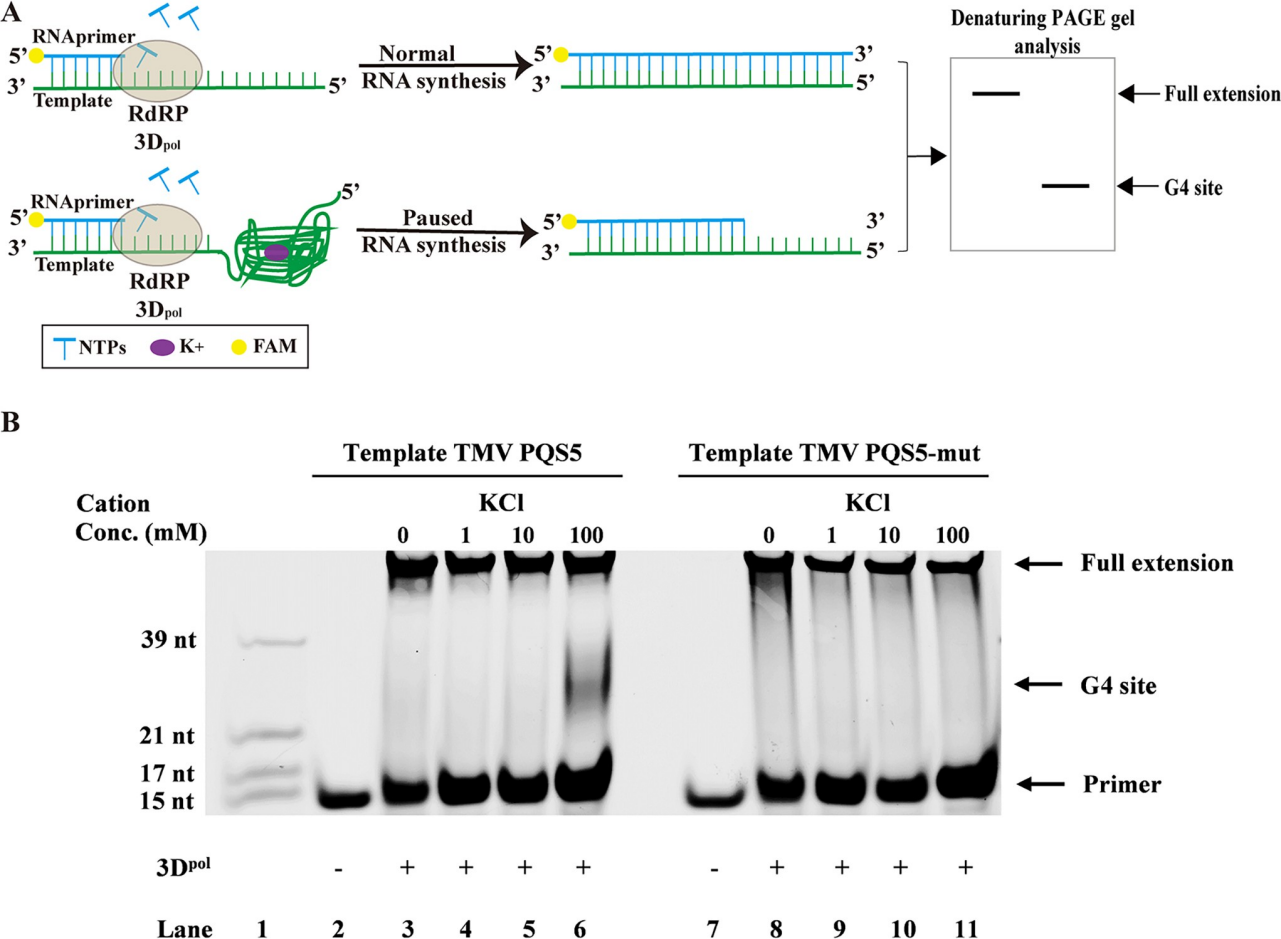
G4 Formation by PQS5 interferes with RNA synthesis *in vitro*. (A) Workflow of RNA stop assay. (B) Denaturing PAGE of the extended RNAs. Lane 1, FAM labeled RNA markers (S2 Table); lane 2 and lane 7, negative control without RdRP 3D^pol^; lane 3 to 6, RNA synthesis with template TMV PQS5 in absence or presence of potassium at 1 mM, 10 mM, 100 mM; lane 8 to 11, RNA synthesis with template TMV PQS5 mutant in absence or presence of potassium at 1 mM, 10 mM, 100 mM.

RNA synthesis is a crucial step in the genome replication of RNA viruses [16]. RNA-dependent RNA polymerase (RdRP) stops at the G4 site in the RNA template, disrupting the new RNA synthesis [17]. To investigate TMV PQS5 functions in RNA synthesis, the RNA stop assay was performed with an RNA template harboring TMV PQS5 sequence in the absence or presence of potassium (Fig 3A). Potassium is a monovalent cation that can induce the formation and stabilization of G4 structure [18]. In the absence of potassium, the TMV PQS5 did not pause RdRP 3D^pol^ (Fig 3B, lane 3). However, when potassium concentration increased to 100 mM, the short RNA fragment at the G4 site occurred (Fig 3B, lane 6), which resulted from the folding of TMV PQS5 in the template strand (S4 Fig). The template with TMV PQS5 mutant (S3 Table) did not respond to potassium and only produced the full extension RNA (Fig 3B, lanes 8 to 11). These results indicated that the folding of TMV PQS5 G4 structure disrupted RNA synthesis.

### 2.3 Folding of TMV PQS5 inhibits TMV proliferation in *N*. *benthamiana*

**Fig 4:**
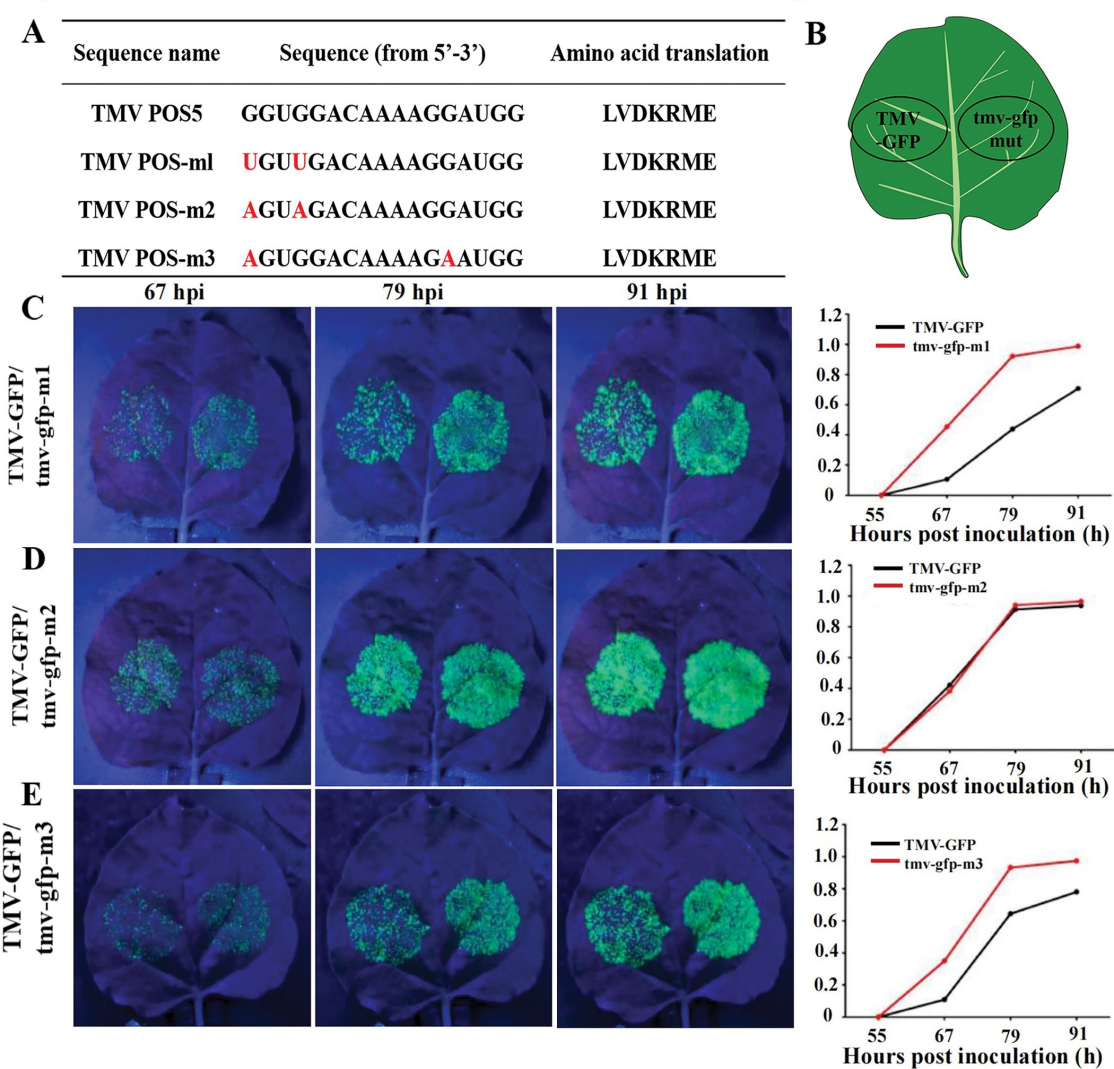
TMV PQS5 folding inhibits GFP expression from TMV genome. (A) Mutant base sites of TMV PQS5 in different expression vectors. (B) Diagram of inoculation of TMV–GFP and tmv–gfp-mut. (C-E) Fluorescence signals of different expression vectors [(C) TMV–GFP and tmv–gfp-m1, (D) TMV–GFP and tmv–gfp-m2, and (E) TMV–GFP and tmv-gfp-m3] in leaves at 67, 79, and 91 hours post-inoculation (hpi) and corresponding plots of the fluorescence area changes with the prolong infiltration time. The ordinate values were derived from the ratio of the actual fluorescence area at different hours post inoculation to the maximum fluorescence area of the corresponding mutant strain.

Based on the aforementioned results, the effect of TMV PQS5 on TMV genome proliferation in plant was evaluated by constructing a transient expression vector of TMV–green fluorescent protein (GFP) (S5 Fig), where fluorescence signals visually reflected TMV genome replication [19]. We constructed three mutant viral expression vectors based on the TMV–GFP vector, namely tmv–gfp-m1, tmv–gfp-m2, and tmv–gfp-m3, in which the synonymous mutants based on TMV PQS5 were designed to ensure the translation of the codons into the same protein (Fig 4A). Therefore, the effect of G4 formation on TMV genome translation could also be reflected by the difference in the fluorescence signal between wild-type TMV and TMV PQS5 mutants (mutants without PQS5).

In this study, the wild-type TMV–GFP was inoculated in the left-half blade of *N*. *benthamiana* leaves, and the PQS5 mutants were inoculated in the right-half blade via *Agrobacterium*-mediated infiltration (Fig 4B). The fluorescence signals of GFP were automatically monitored and quantified continuously for 150 h after infiltration. At the same hour post-inoculation, the fluorescence signals of leaves treated with tmv–gfp-m1 and tmv–gfp-m3 were markedly stronger than those of the leaves treated with the wild-type TMV–GFP (Fig 4C–4E). The results suggested that the TMV PQS5 inhibited TMV proliferation. However, the plot of the change in fluorescence area of tmv–gfp-m2 was consistent with that of the wild-type TMV–GFP. This might be owing to the low translation efficiency of new codons, interference due to a new secondary structure, or other nonspecific interactions, which counterbalances the positive regulation from G4 mutants.

### 2.4 TMV PQS5 ligand NMM inhibits TMV replication and gene expression *in vivo* and exhibits antiviral activity

**Fig 5:**
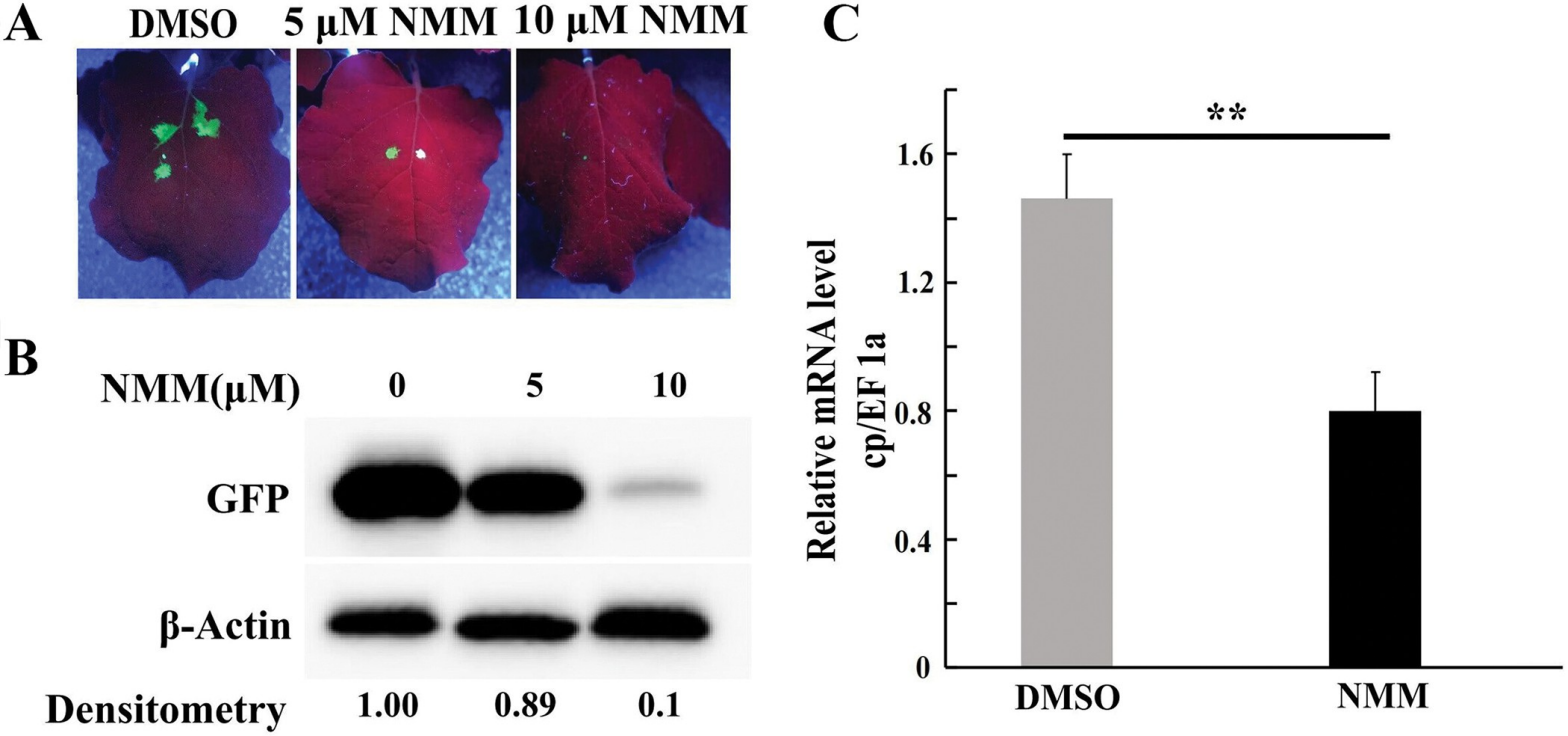
NMM exhibits strong inhibitory effects against TMV. (A) Green fluorescence of TMV-GFP in *N*. *benthamiana* leaves treated by NMM at 5 μM, 10μM. (B) Western blot analysis for GFP expression in the *N*. *benthamiana* leaves treated by NMM at 5 μM, 10 μM. (C) TMV genomic RNA level in the *N*. *benthamiana* leaves treated by 50 μΜ NMM. Relative mRNA level of TMV *cp* gene was detected by qRT-PCR with the cDNA that was reverse transcript from the genomic RNA with TMV specific primer.

We investigated the effect of NMM on the replication of TMV–GFP infectious clones in *N*. *benthamiana* by using TMV–GFP expression vectors through digital fluorescence visual screening and half-leaf comparison method [20]. Different concentrations of NMM (S6 Fig, 5 nM, 500 nM, 5 μM) were mixed with TMV–GFP infectious clones and infiltrated into the leaves. Of note, NMM at 5 μM exhibited a stronger inhibitory effect against TMV than the commercial antiviral ningnanmycin at 560 μM, which was reflected from the fluorescence area of GFP (S7 Fig). GFP expression was downregulated in a dose dependent manner when 5 μM or 10 μM NMM was infiltrated into the leaves (Fig 5A and 5B). The amount of TMV *cp* transcript is generally used as an indicator to determine the expression level of TMV [21]. The relative mRNA levels of *cp* in the NMM (50 μM)-treated samples decreased by 40% compared with those of the control (Fig 5C and S4 Table). Taken together, our results suggested the potential activity of G4 ligands as antivirals.

The activity of NMM was evaluated using half-leaf methods in three modes (inactivation effect, protection effect, and curative effect) [20]. The results showed that the antiviral activity of NMM was superior to that of the commercial antiviral agent ribavirin, and its curative effect was better than the inactivation and protection at 500 mg/L (S5 Table). When directly injected into leaves, NMM showed stronger antiviral activity than ningnanmycin (S7 Fig). However, its activity *in vivo* was weaker than that of ningnanmycin, suggesting that the absorption and conduction efficiency of small molecules in plants should be improved.

Classic G4 ligands, namely TMPyP4 and BRACO-19 (S6 Fig), were identified to interact with RNA TMV PQS5 by analyzing the change in the UV and fluorescence spectra of the compounds with the addition of RNA (S8 Fig). Both BRACO-19 and TMPyP4 at 10 μM exhibited moderate antiviral activity; however, the leaves treated with TMPyP4 showed necrosis, indicating that 10 μM TMPyP4 was phytotoxic to tobacco (S9 Fig). Another two plant isoquinoline alkaloids, Sanguinarine and Tryptanthrin (S6 Fig), also showed interaction with TMV PQS5 (S10 Fig). However, their low solubility limited further development. These results suggested that plant endogenous compounds may inhibit virus proliferation in plants by interacting with G4s, even though their inadequate permeability limits their antiviral potential.

### 2.5 Screening for the photosensitive molecules binding with TMV PQS5

**Fig 6:**
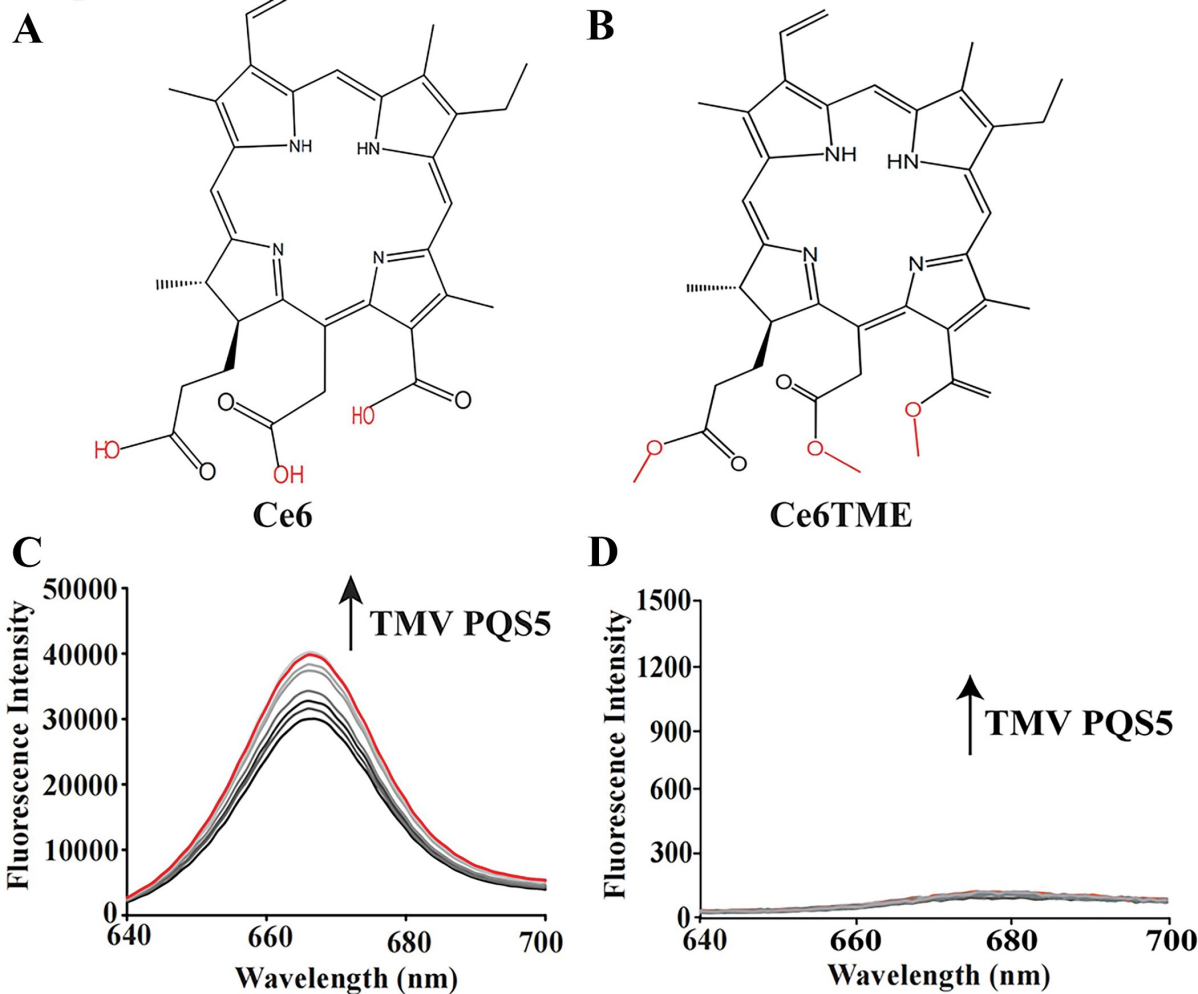
Photosensitive Ce6 and Ce6TME can bind with TMV PQS5. Chemical structures of Ce6 (A) and Ce6TME (B). Fluorescence responses of Ce6 (C) and Ce6TME (D) (1.5 μM) to increasing amounts of TMV PQS5 from 0 to 8 μM at 25°C. The spectra of the mixture with 8 μM TMV PQS5 and 1.5 μM Ce6 were highlighted in red, λ_ex_ = 405 nm.

The photosensitivity of some G4 ligands enables the study to be more attractive [22]. When a PS binds to the G4 in the viral genome, the emitted ROS under light can preferentially break the nucleotides and inhibit the proliferation of plant viruses. In this study, commercially available PSs (S11 Fig) were tested for their ability to bind with TMV PQS5, and their anti-TMV activities in vivo were also detected (S7 Table). With the titration of TMV PQS5, the absorbance spectrum of chlorin Ce6 increased immensely (Figs 6A and S12), and the fluorescence intensities of Ce6 also increased (Fig 6C). On the basis of fluorescence titrations, the *K*_*d*_ was derived as 0.93 μM, indicating a strong interaction between Ce6 and TMV PQS5. Ce6TME, an ester-substituted derivative of chlorin Ce6 (Fig 6B), exhibited only a slight increase in the absorbance spectra (S12 Fig), and no distinct changes were observed in its fluorescence spectrum (Fig 6D), indicating that the peripheral substituents of chlorins affected the binding with the G4 structure. The strong binding between Ce6 and TMV PQS5 was further validated by the increment in the CD spectrum signals of TMV PQS5 with the addition of Ce6 (S13 Fig).

### 2.6 Ce6 cleaves TMV PQS5 upon photo-irradiation

#### 2.6.1 Ce6 generates ROS *in vitro* and in BY-2 cells upon photoirradiation

**Fig 7:**
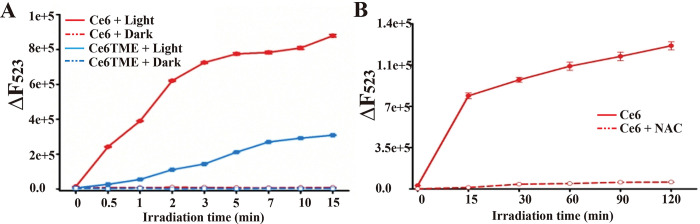
ROS generated from the solution with Ce6 upon photoirradiation. (A) Fluorescence changes in solution of 10 μM DCFH-DA in the presence of 10 μM Ce6 or Ce6TME with or without light irradiation at 25°C. Fluorescence signals of 10 μM DCFH-DA works as an ROS indicator. (B) Fluorescence intensity changes of 10 μM Ce6 in the absence or presence of 10 mM NAC upon photoirradiation at 25°C. λ_ex_ = 488 nm, λ_em_ = 523 nm.

PSs produce ROS when exposed to light that damages nearby nucleic acids or proteins, thereby killing pathogens [23]. Dichloro-dihydro-fluorescein diacetate (DCFH-DA) is widely employed as a ROS indicator to detect the newly generated ROS, in which the non-fluorescent DCFH is oxidized to produce highly fluorescent DCF in the presence of ROS (S14 Fig). Herein, the fluorescence intensity in the solution is proportional to the concentration of ROS. The results showed that the ROS yielded by Ce6 and Ce6TME increased significantly with the prolonged irradiation time either in solution or in the BY-2 cells (Figs 7A and S15). The speed of ROS yielded by Ce6 was 3–4 times higher than that achieved by using Ce6TME (Fig 7A). The fluorescence intensity decreased significantly after the addition of a ROS scavenger, namely N-acetyl-L-cysteine (NAC) (Figs 7B and S16).

#### 2.6.2 Ce6 cleaves TMV PQS5 with selectivity upon photoirradiation

**Fig 8:**
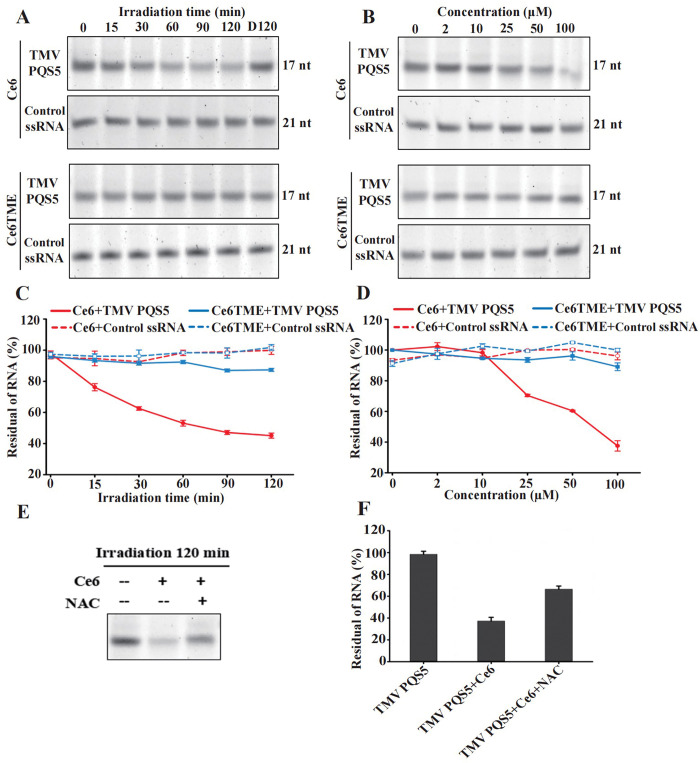
Ce6 decreases the amount of TMV PQS5 upon photo-irradiation. Denaturing polyacrylamide gel electrophoresis (20%) of 0.3 μM TMV PQS5 or the ssRNA control in the presence of 100 μM Ce6, Ce6TME after photo-irradiation for the indicated periods (0, 15, 30, 60, 90, 120 min) at 25°C (A), and in the presence of Ce6, Ce6TME with different concentrations (0, 2, 10, 25, 50, and 100 μM) after photo-irradiation for 120 min at 25°C (B). The lane of “D120” means that the samples were treated under dark for 120 min. Change of residual intact RNA of TMV PQS5 or Control ssRNA with the increasing irradiation time (C), and change of residual intact RNA of TMV PQS5 or Control ssRNA with compounds at different concentrations (0–100 μM) (D). Error bars represent mean ± SD; n = 3. (E) 20% denaturing polyacrylamide gel electrophoresis of 0.3 μM TMV PQS5 mixing with 100 μM Ce6 in the absence or presence of 10 mM NAC, followed by photo-irradiation for 120 min at 25°C. (F) Residual intact TMV PQS5 RNA from denaturing polyacrylamide gel after photo-irradiation. Error bars represent mean ± SD; n = 3.

The effect of Ce6 on TMV PQS5 upon photoirradiation was analyzed using the denaturing polyacrylamide gel electrophoresis (Fig 8). In the presence of Ce6, the band intensity of TMV PQS5 gradually decreased with extended irradiation time (Fig 8A), and only 45% of TMV PQS5 was left after irradiation for 120 min (Fig 8C). The cleaved product of TMV PQS5 was then observed (S17A Fig). The cleavage of TMV PQS5 treated with Ce6TME was negligible even after irradiation for 120 min. Furthermore, TMV PQS5 was photocleaved by Ce6 in a dose-dependent manner (Fig 8B and 8D). In the absence of Ce6, the TMV PQS5 in the buffer was stable, and no detectable cleavage was detected under the same irradiation conditions (S17B Fig). Ce6 did not cleave TMV PQS5 without photoirradiation, as shown in the lane of “D120” (Fig 8A). The cleavage of the control ssRNA (a non-G4-forming sequence, the detail is mentioned in Materials and Methods section 4.10) by Ce6 was negligible under the same conditions (Fig 8), indicating that G4 was more vulnerable than the ssRNA control. The addition of NAC decreased the photocleavage of TMV PQS5 by Ce6 (Fig 8E and 8F), indicating that the photocleavage of Ce6 to TMV PQS5 is dependent on ROS generation.

To further investigate the dependence of the cleavage on Ce6 or photo-irradiation, mass spectrometry was employed to analyze the RNA fragments from the solution with TMV PQS5 and Ce6 (S18 Fig). The addition of Ce6 to TMV PQS5 under the dark conditions did not result in the cleavage of the G4-forming sequence (Fig 8A), and the base peak in the mass spectrum remained the intact PQS5 sequence (S18C Fig). Similarly, in the case of TMV PQS5 without Ce6 under the LED white light (55 W) for 120 min, RNA fragments were still observed with the base peak (S18B Fig). After adding Ce6 (100 μmol/L) and exposing the solution to the LED white light for 120 min, the intact PQS5 sequence was not identified in the mass spectrum of the annealed TMV PQS5; however, dozens of RNA fragments were observed (S18A Fig). Thus, these results indicated that both Ce6 and photo-irradiation are crucial for the cleavage of PQS.

### 2.7 Upon photoirradiation, Ce6 suppressed TMV expression with ROS dependency

The effect of Ce6 on the expression of TMV RNA was further evaluated by qRT-PCR. In this assay, BY-2 protoplasts pre-infected with TMV were incubated with Ce6 at different concentrations, followed by exposure to light or dark conditions. Afterwards, the total RNA from BY-2 protoplasts was extracted, and the cDNA was prepared for qRT-PCR analysis.

**Fig 9:**
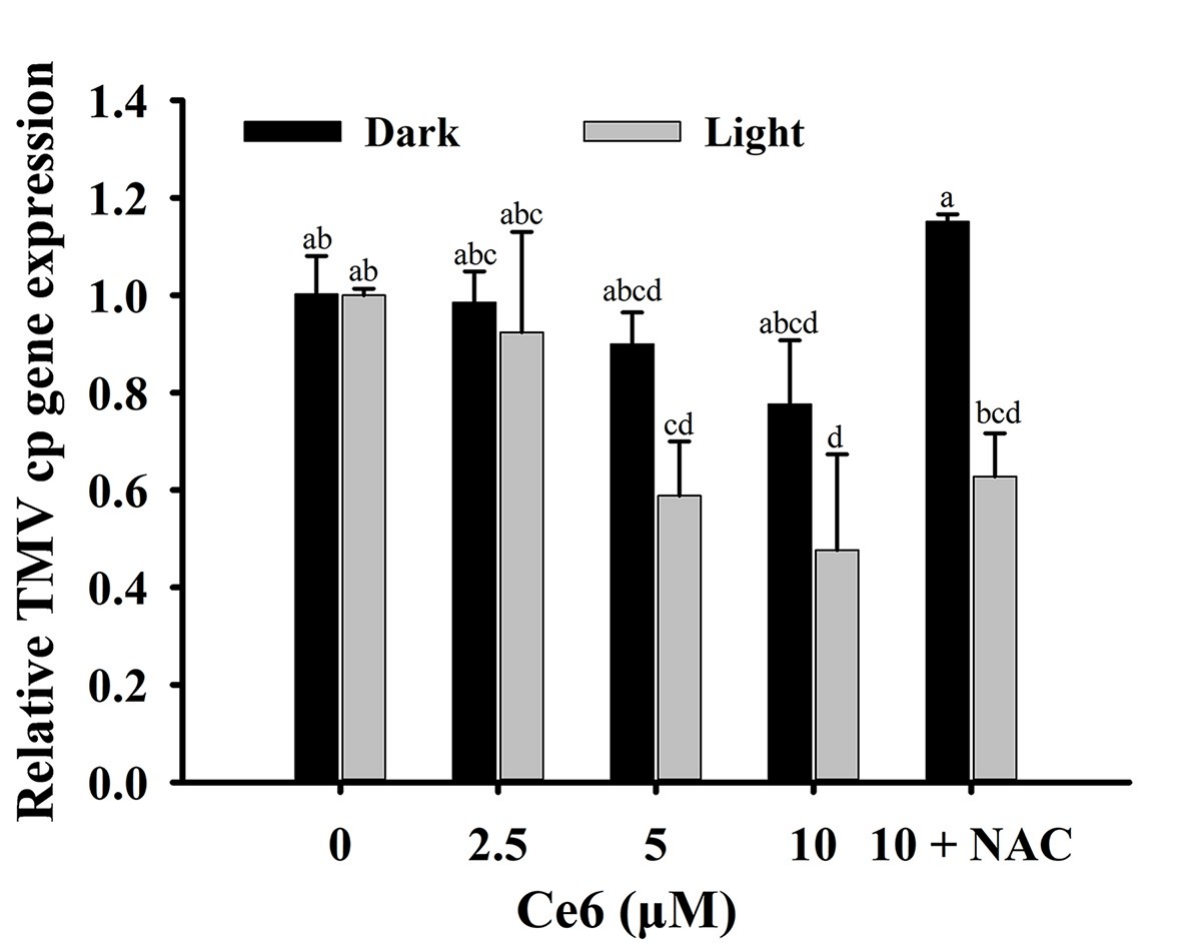
QRT-PCR assay of relative expression of the TMV *cp* gene in BY-2 cells. BY-2 cells were pre-infected with TMV virions for 3 h, and then treated with Ce6, followed by being irradiated with white light or in the dark for 0.5 h. In the treatment of “10 + NAC”, BY-2 cells infected with TMV virions were treated with 10 μM Ce6 and 10 mM NAC.

The relative expression of the TMV *cp* gene decreased with the increase in Ce6 concentration (Fig 9). Under dark conditions, 10 μM Ce6 exhibited a weak inhibitory effect against TMV, and the relative expression levels of the TMV *cp* gene decreased to 77.6%. Upon photoirradiation, the number of viral RNA copies decreased significantly to 47.7%. Furthermore, when BY-2 cells were treated with Ce6 and NAC, the inhibitory effect of Ce6 against TMV was alleviated, and the relative expression of the TMV *cp* gene was 62.8% after light irradiation, suggesting that NAC weakened the anti-TMV activity of Ce6. These results further indicated that Ce6 effectively suppressed the expression of TMV RNA upon photoirradiation.

### 2.8 Ce6 exhibits higher anti-TMV activity *in vivo* upon photoirradiation

We further investigated the anti-TMV activity of Ce6 under LED-light by using the lesion method (Table 1). Under normal-light irradiation, Ce6 inhibited 30.0% of the virus at 100 μg/mL, and exhibited an inhibition rate of about 78.1% at 500 μg/mL, which were higher than those of commercial ningnanmycin (with inhibition rates of 36.3% and 76.6% at 100 μg/mL and 500 μg/mL, respectively). Under LED-light irradiation, Ce6 exhibited higher activity against TMV with inhibition rates of 51.8% at 100 μg/mL, and 92.2% at 500 μg/mL, which were significantly higher than those of ningnanmycin (inhibition rates of 39.5% and 80.7% at 100 μg/mL and 500 μg/mL, respectively).

**Table 1:** Anti-TMV activity of Ce6 under normal-light and LED-light irradiation *in vivo*.

Compds.	Conc. (μg/mL)	Inactivating activity (%)	
		Normal-light irradiation	LED-light irradiation
Ce6	50	7.3 ± 5.9	10.3 ± 3.3
	100	30.0 ± 2.4	51.8 ± 8.1
	200	50.4 ± 6.1	68.0 ± 8.0
	500	78.1 ± 9.6	92.2 ± 3.1
Ningnanmycin	100	36.3 ± 5.0	39.5 ± 7.5
	500	76.6 ± 1.9	80.7 ± 3.2

### 2.9 Ce6 inhibits TMV proliferation via binding to RNA G4 in the viral genome

**Fig 10:**
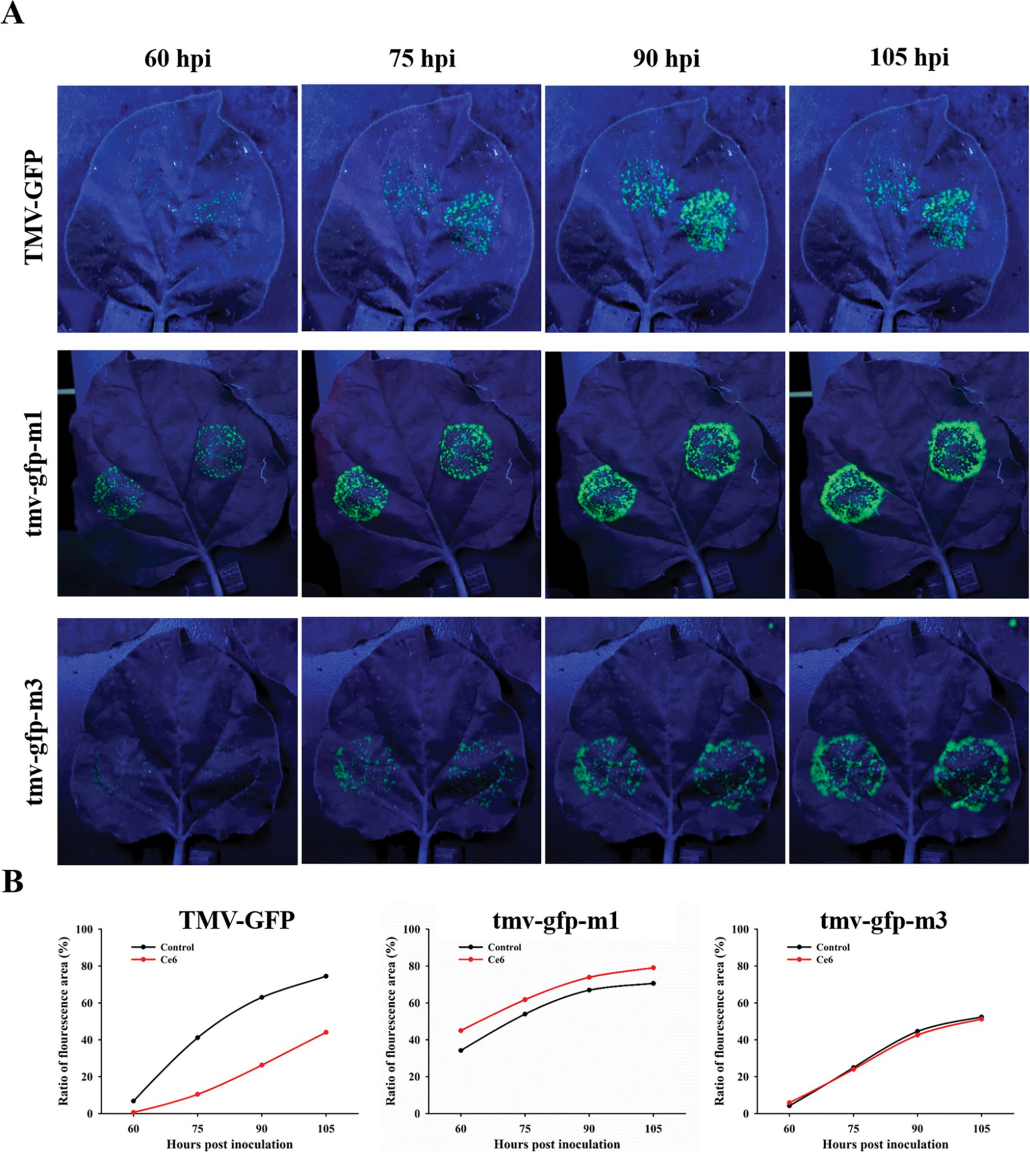
Ce6 inhibits TMV proliferation via binding to RNA G4 in viral genome. (A) Fluorescence signals of different expression vectors in leaves treated with 10 μM Ce6 in left-half and 1% DMSO in right-half at 60, 75, 90, and 105 hpi. (B) Plots of the fluorescence area changes of different expression vectors with/without Ce6 at different hpi.

Among the three constructed G4 synonymous mutant strains, two strains proliferated significantly faster than the wild strain (Fig 4), indicating that the formation of G4 interfered with virus proliferation. In this study, we investigated the effect of Ce6 on the proliferative rate of wild-type and two mutant strains in tobacco to determine whether small molecules acted on G4. The left-half of the leaves was infiltrated with the mixtures of viral expression vectors and Ce6, and the right-half blades were infiltrated with the mixtures of the same expression vector and DMSO. For all three viral expression vectors, the fluorescence area increased with the prolonged infection time (Fig 10). For the wild-type strain, the addition of Ce6 decreased the virus proliferation rate; however, Ce6 did not decrease the proliferation rate of the mutant strains. This result suggested that the inhibitory effect of Ce6 was dependent on the existence of G4 structures in the viral genome.

### 2.10 Chlorins showed no phytotoxicity in plants or BY-2 cell line

When the tobacco plants were sprayed with Ce6 at 500 μg/mL for 4 days, no phytotoxicity or side effect was observed (S19 Fig). The effect of Ce6 on the metabolic activity of BY-2 cells was evaluated by performing the resazurin assay. In this assay, non-fluorescent resazurin is taken up by viable cells and metabolized to fluorescent resorufin by several different redox enzymes in cells, and the fluorescence changes of resorufin reflect the effects of chlorine on redox enzymes [24]. After BY-2 cells were treated with Ce6, their metabolic activities were maintained at around 80% even with 100 μM of Ce6 (S20 Fig). These results suggested that Ce6 showed no phytotoxicity to plants and did not affect the metabolic activity of BY-2 cells.

## 3. Discussion

Folding or unfolding of G4 regulates multiple biological and cellular processes. Investigating the biological regulatory network centering on G4s will provide information to elucidate the confrontation mechanism between the hosts and the viruses. In this study, we identified a stretch of RNA sequences PQS5 in the movement protein ORF of TMV genome, which forms G4 *in vitro* (Figs 1 and 2). The G4 structure inhibited a viral RNA dependent RNA polymerase activity in an *in vitro* assay (Fig 3). During TMV replication, its genomic RNA serves as the template for synthesis of minus strand viral RNA, which in turn serves as the template for synthesis of viral genomic or subgenomic RNAs. Thus, our results indicated that PQS5-G4 plays a negative role in the negative strand viral RNA synthesis during viral replication. This conclusion was further validated using half-leaf comparison assays. The synonymous mutations changing the guanine nucleotides involving G4 formation promoted viral proliferation in plants (Fig 4). These indicated that PQS5 could form a G4 structure *in vivo* and regulate viral replication, which will open a new avenue in plant-virus interaction studies.

The driving force of folding or unfolding is not only the G4-forming sequences themselves but also the biomolecules in organisms [25]. Recently, multiple metabolites or proteins derived from viruses or hosts have been reported to interact with G4s to regulate viral life cycles [26], suggesting that G4s might function as hubs in the arm-wrestle competition between viruses and plants, and the plant metabolites or proteins may trigger these processes [27, 28]. NMM and some plant secondary metabolites interacted with G4 in the TMV genome, and could work as the antivirals (Fig 5). Thus, plant viruses can be prevented and controlled by synthesizing chemicals targeting the functional G4, or breeding disease-resistant cultivars by regulating the contents of G4 binders in crop metabolites.

Nowadays, PSs have been widely reported and are considered for medicinal application against cancer [29–31]; In the presence of light and oxygen, PSs catalyze the production of monomer oxygen to inhibit pests, and the quantum yield of singlet oxygen determines their anti-pest activity [32]. PSs act as catalysts without toxic reactions and easily degradation without polluting the environment. However, unwanted damage to healthy tissues can be a key problem that limits the use of PSs. Thus, guiding PSs to gather around the targets is crucial to achieving selectivity [33]. In this study, we screened commercially available PSs that can bind with TMV PQS5-G4 (Fig 6). Ce6, one of the PQS5-G4 binding small molecules, mainly photocleaved G4 with selectivity by producing ROS (Figs 7 and 8). Thus, we then evaluated the antiviral effects of Ce6 against TMV. Ce6 exhibited higher anti-TMV activity *in vivo* and in BY-2 cells upon photoirradiation (Fig 9 and Table 1), and inhibited viral GFP accumulation from wild-type but not PQS5 mutant viruses (Fig 10). To the best of our knowledge, our study is the first reported an antiviral PS by targeting G4 structures in the plant virus genome. Our results suggest that the application of PSs designed to target the specific functional components of plant viruses is a novel strategy to control plant viruses.

## 4. Materials and methods

### 4.1 Materials and chemicals

RNA oligonucleotides. All the high-pressure liquid chromatography (HPLC)-grade RNA oligonucleotides used in this study were purchased from Sangon Biotech (Shanghai) Co. Ltd., and used without further purification. Single strand concentrations of the oligonucleotides were determined by measuring the absorbance at 260 nm using a UV-1800 spectrometer (Shimadzu Technologies, Japan). Oligonucleotide solutions (100 μM) in Diethyl pyrocarbonate (DEPC)-treated water were stored at -80°C. The oligonucleotides were diluted into 10 μM in phosphate buffer containing 10 mM phosphate (pH 7.0) and 100 mM KCl, and heated at 90°C for 5 min, then annealed to room temperature overnight.

Chemicals. *N*-Methyl Mesoporphyrin IX (NMM, CAS: 142234-85-3) and Meso-Tetra (*N*-Methyl-4-pyridyl) porphine tetrachloride (TMPyP_4_, CAS: 92739-63-4) was purchased from Santa Cruz Biotechnology, Inc. (Dallas, Texas, U.S.A.). BRACO-19 (CAS: 351351-75-2, B19) were purchased from Sigma-Aldrich, Inc. (St. Louis, MO, United States). Chlorin e6 (CAS: 19660-77-6, named Ce6), pyropheophorbide-a (CAS: 24533-72-0, named PP-a), chlorin e6 trisodium (CAS: 72984-36-2, Ce6T), and chlorin e6 trimethyl ester (CAS: 35038-32-5, Ce6TME) were purchased from Frontier Scientific (Newark, DE, U.S.A.); Hematoporphyrin dihydrochloride (CAS:17696-69-4, named HD) were purchased from J&K Scientific Ltd.; Protoporphyrin IX (CAS: 553-12-8, named PPIX), protoporphyrin IX dimethyl ester (CAS: 5522-66-7, named PPIXDME), mesoporphyrin IX dihydrochloride (CAS: 68938-72-7, named MPCl), Zn(II) Protoporphyrin IX (CAS: 15442-64-5, named Zn(II)-PPIX), rose bengal (CAS: 632-69-9, named RB), and 9-Aminoacridine hydrochloride monohydrate (CAS: 52417-22-8, named 9AA-HCl) were brought from Sigma-Aldrich, Inc. (St. Louis, MO, United States).Sanguinarine (CAS: 2447-54-3), Nitidine Chloride (CAS: 13063-04-2) and Tryptanthrin (CAS: 13220-57-0) were purchased from MedChemExpress LLC (Shanghai, China).

Biological materials. Tobaccos *Nicotiana tabacum var Xanthi nc* and *Nicotiana*. *benthamiana* (*N*. *benthamiana*) were cultivated in the greenhouse and kept under the conditions of 26 ± 2°C and 60 ± 2% relative humidity with light/dark cycles of 14/10 h. Tobacco grew to the 5–6 leaf stage was selected. The suspensions of tobacco cells (Tobacco Bright Yellow 2, BY-2) were cultured in the dark condition with continuous agitation (130 rpm) at 26°C. The suspensions were growing in the liquid Murashige and Skoog medium (pH 5.8) supplemented by 2,4-dichlorophenoxyacetic acid (0.2 μg/mL) and thiamine (0.2 μg/mL) solutions. BY-2 cell suspensions were subcultured every 7 days by transferring 5 mL aliquot into 50 mL of fresh medium. Exponential growth phase cells (3 or 4 days after dilution) were used for the experiments.

### 4.2 Circular dichroism

Circular dichroism (CD) spectra were performed with the JASCO 1500 spectropolarimeter (JASCO, Japan) with a 1 mm quartz cuvette at 25°C under a constant flow of nitrogen. 15 μM pre-annealed TMV PQS5 in solution with KCl at different concentrations (0, 1, 50, 100 mM) were detected to the formation of G4. The mixtures of 15 μM pre-annealed TMV PQS5 and 80 μM compounds were incubated at 30°C for 10 min, and then, the CD spectra were recorded covering a spectral range of 200−350 nm using a scan rate of 200 nm/min with a 1 s response time and 1 nm bandwidth.

### 4.3 Nuclear magnetic resonance (NMR) spectroscopy

The 1D ^1^H NMR spectroscopy experiment for RNA PQS5 was recorded at 318K on a Bruker 700 MHz (BioSpin, Germany). TMV PQS5 at a concentration of 360 μM containing 5 mM Kcaco, and 200 mM KCl were heated to 95°C for 5 min, and then gradually cooled to room temperature. The NMR experiments for samples in water solution were performed with Watergate or Jump-and-Return water suppression techniques. The mixing time was 300 ms. In 1D experiments, NMR spectra were processed and visualized by MestReNova (mestrelab.com). A total of 512 scans were acquired for spectrum of TMV PQS5 with a relaxation delay of 2 s between scans.

### 4.4 RNA stop assay

The assay was performed as described previously with some modifications [34]. RNA primer 15 (3 μM) and template RNA (TMV PQS5 or TMV PQS5-mut, 6 μM) were annealed in 10 mM phosphate buffer pH 7.0 supplemented with KCl at different concentrations (0.1 mM, 10 mM, 100 mM) by heating at 95°C for 5 min and being cooled down to room temperature slowly. Then 1 μL annealed sample was added into 19 μL reaction buffer (50 mM HEPES pH 7.0, 20 mM NaCl, 5 mM MgCl_2_ and 4 mM DTT) with 3D^pol^ (0.2 mg/reaction) and NTPs (200 μM), and the mixture was incubated at 33°C for 60 min. The reactions were stopped by adding an equal volume of 95% formamide and being heated at 90°C for 4 min. The products were separated through electrophoresis on 20% denaturing polyacrylamide gels. The gels were scanned by the Amersham Typhoon 5 (Cytiva, United States) in fluorescence mode. The sequences used in this assay were listed in S3 Table.

### 4.5 Plasmid construction

Three TMV-GFP vectors with synonymous mutant of TMV PQS5 sequence were constructed using empty vector pH7LIC1.0 and viral vector pH7LIC14−TMV−GFP. Briefly, pH7LIC1.0 was digested through restriction enzyme SmaI (R0141, NEB) to obtain linearized vector. The sequence of TMV at 1058−4845 bp was amplified using two pairs of primers TMM076/TMM077 based on the template pH7LIC14−TMV−GFP. The purified PCR products were cloned into the linearized pH7LIC1.0 to obtain the intermediate vector using homologous recombinant enzyme (C112, Vazyme), named pH7LIC1.0−TMV−GFP-step1. And then the pH7LIC1.0−TMV−GFP-step1 was digested by restriction enzyme HindIII (R3104, NEB). Seven sequences (named s1, s2-1, s2-2, s2-3, s3-1, s3-2, and s3-3) were amplified using the specific primers (TMM081/TMM083 for s1, TMM082/TMM022 for s2-1, TMM082/TMM030 for s-2-2, TMM082/TMM032 for s2-3, TMM080/TMM021 for s-3-1, TMM080/TMM029 for s-3-2, and TMM080/TMM031 for s-3-3) based on the template of pH7LIC1.0−TMV−GFP-step1, and then, they were connected by fusion PCR in sequence to obtain the intermediate mutant vectors, named pH7LIC1.0−tmv−gfp-m1, pH7LIC1.0−tmv−gfp-m2, and pH7LIC1.0−tmv−gfp-m3. Therein, pH7LIC1.0−tmv−gfp-m1 were fused by the sequence amplified using s2-1 and s3-1, pH7LIC1.0−tmv−gfp-m2 were fused by the sequence amplified using s2-2 and s3-2, and pH7LIC1.0−tmv−gfp-m3 were fused by the sequence amplified using s2-3 and s3-3. The pH7LIC14−TMV−GFP was digested through restriction enzyme ApaI (R0114, NEB) and StuI (R0187, NEB) to obtain linearized vector. The sequences of TMV at 1058−4845 bp were amplified using primer pair TMM015/TMM016 and the three intermediate mutant vectors as templates. The purified PCR products were cloned into the linearized pH7LIC14−TMV−GFP. Finally, the identified positive clone plasmids with correct sequences were electroporated into *Agrobacterium tumefaciens* according to our previous report, and the mutant vectors were named as tmv−gfp-m1, tmv−gfp-m2, and tmv−gfp-m3, respectively. All primers used for constructing synonymous mutants were listed in S6 Table.

### 4.6 Western blot

NMM at different concentrations was mixed with TMV−GFP infectious clone for 30 min. Subsequently, the mixtures were injected into *N*. *benthamiana* leaves, in which the inoculation volume was 200 μL and diameter kept at 2 cm. Proteins delineated in the infection areas of the leaves were harvested and lysed with SDS-PAGE loading buffer. Western blot was performed according to the manufacturer’s instructions. Mouse monoclonal GFP antibody and β−actin antibody were purchased from Beyotime, China. The relative expression levels of GFP in the *N*. *benthamiana* leaves treated by NMM (5 μM and 10 μM) were analyzed via Image J software. The *N*. *benthamiana* leaves treated by the same volume of DMSO served as the controls.

### 4.7 Quantitative real-time PCR

Effect of NMM on the gene expression of TMV genome *in vivo*. Different concentrations of NMM were mixed with TMV−GFP infectious clone and injected into the leaves as described above. The delineated infection areas of leaves were collected and used for subsequent trials.

Effect of Ce6 on the expression of TMV genome in BY-2 cells upon photo-irradiation. BY-2 cells (3-day after subcultures) were treated with an enzymatic hydrolysis buffer (10 mg/mL cellulose RS and 1 mg/mL pectolyase Y23 in 0.4 M mannitol) for 3−4 h to remove the cell wall components. The BY-2 protoplast precipitates were obtained by centrifuging and removing the supernatant. BY-2 protoplasts were infected with TMV virions and incubated for 3 h on ice, then serially diluted solutions of Ce6 (2.5, 5, and 10 μM) were added and maintained in the dark for 0.5 h. Subsequently, the mixtures were photo-irradiated or under dark for 0.5 h, washed three times with 0.4 M mannitol buffer, and incubated in the buffer (4 mM 2-(*N*-morpholino) ethanesulphonic acid (MES), 20 mM KCl in 0.5 M mannitol) for 16 h. The BY-2 protoplasts were collected and used for subsequent trials.

The total RNAs were extracted using TRIzol reagent (Takara Bio Inc.) according to the manufacturer protocols. Single-stranded cDNA of the samples were synthesized using the PrimeScript RT reagent Kit (Takara Bio Inc.). Real-time PCR reactions were performed on a Quant Studio 3 PCR System (Applied Biosystems, Life Technologies, USA). The conditions of each amplification reaction were as follows: 95°C for 30 s, then 40 cycles of 95°C for 5 s and 57°C for 34 s, 95°C for 15 s and 60°C for 60 s. The primers used were listed in S4 Table. The expression level of TMV coat protein (*cp*) gene was normalized using *N*. *benthamiana* Elongation Factor 1-alpha (Nb EF1a) gene as a reference gene, and the results were analyzed with the 2^−ΔΔCt^ method. Each sample was subjected to three independent replicates. All assays were performed with three independent biological replicates.

### 4.8 UV-Visible absorption and fluorescence spectroscopy

UV-Visible (Vis) absorption spectroscopy. UV-Vis absorption spectra were collected on a UV1800 spectrophotometer (Shimadzu Technologies, Japan) with a quartz cell with 1 mm path length under dark condition. The mixtures of 4 μM pre-annealed TMV PQS5 and 2 μM compounds were incubated at 30°C for 10 min, and then, the UV-Vis spectra of samples were recorded covering a spectral range of 200−800 nm.

Fluorescence spectroscopy. Fluorescence titration spectra were recorded on a RF-5301PC spectrofluorometer (Shimadzu Technologies, Japan) with a quartz cell with 1 mm path length under dark condition. For the fluorescence titration of compounds with TMV PQS5, the concentration of each compound was fixed at 1.5 μM, and titrated with increasing amounts of pre-annealed TMV PQS5. After incubation at 30°C for 10 min, the fluorescence spectra of samples were recorded covering a spectral range of 500−800 nm using a scan rate of 6000 nm/min. The excitation wavelength of the chlorins was 405 nm.

Measurement of dissociation constant (*K*_*d*_). The dissociation constant (*K*_*d*_) of compounds to TMV PQS5 were calculated from the fluorescence titration by linear curve fitting according to the following equation [35]:

$$log\frac{F(\left[M\right])-F_{min}}{F_{max}-F([M])}=nlog[M]-logK_{d}$$

where F_min_ is the fluorescence emission intensity collected at 665 nm for the free compound; F_max_ is the maximum fluorescence intensity upon saturation; F denotes the fluorescence intensity in the presence of TMV PQS5 with different concentrations; n represents the stoichiometric ratio between the compound and TMV PQS5; and [M] represents the concentration of TMV PQS5.

### 4.9 Determination of ROS in aqueous solution and in BY-2 cells

Dichloro-dihydro-fluorescein diacetate (DCFH-DA) was employed to detect the ROS generation [36]. Before the detection of ROS in an aqueous solution, DCFH-DA (10 mM) was dissolved in ethanol, and then was treated by adding 4-fold volume of NaOH (10 mM) for 30 min at room temperature, yielding a nonfluorescent DCFH intermediate. The solution was neutralized with phosphate buffer (shielded with aluminum foil), and the final concentration of DCFH was 10 μM. The mixtures of compounds (10 μM) and DCFH were irradiated with a LED white-light (55W, color temperature of 6500K) or under dark condition. The generation of ROS was detected by monitoring the fluorescence emission of DCF at 523 nm with the excitation wavelength of 488 nm.

Before the detection of ROS in BY-2 cells, cell suspensions (3-day after subcultured) were pre-treated with compounds (10 μM) for 2 h, and then washed with fresh medium to remove the free compounds. DCFH (10 μM) was added into the cell suspensions and incubated for 30 min, followed by washing with fresh medium. After photo-irradiation for 30 min, the cells were visualized using a fluorescence microscope (Olympus BX51, Tokyo, Japan) with a 488 nm laser, and the fluorescence intensity was analyzed under the same contrast.

### 4.10 Denatured polyacrylamide gel electrophoresis analysis and mass spectrometry of RNA oligomers photo-cleavage by chlorins

The mixture of RNA oligomers [TMV PQS5 (sequence: 5’-GGUGGACAAAAGGAUGG-3’) or Control ssRNA (sequence: 5’-UUGUACUACACAAAAGUACUG-3’), 300 nM], and chlorins (100 μM) in phosphate buffer were incubated at 30°C for 10 min. The samples were then irradiated for 0, 15, 30, 60, 90, and 120 min. The sample under dark condition served as control. After photo-irradiation, a 1.5-fold volume of stop solution (80 wt% formamide, 10 mM Na_2_EDTA) was added. And then, the samples with 1.6 pmol RNA were analyzed by electrophoresis in 20% denaturing polyacrylamide gels with 7 M urea. The gels were stained with SYBR Gold, and imaged on a Bio-Rad SYSTEM GelDoc XR^+^. Band intensities were quantified by Image J software. To check the dependence of photo-cleavage on the compound concentration, the mixtures of RNA oligomers (300 nM) and compounds with different concentration (2, 10, 25, 50, and 100 μM) in phosphate buffer were incubated at 30°C for 10 min, and then were irradiated for 120 min.

To further investigate whether the cleavage of TMV PQS5 is correlated with chlorins and photo-irradiation, the liquid chromatography-mass spectrometry (LC-MS) experiments were performed on a Vion quadrupole-time-of-flight (Q-TOF) mass spectrometer coupled with an ACQUITY UPLC I-Class system (Waters, Milford, MA USA). Samples A-C were prepared as follows: (A) The annealed TMV PQS5 (a final concentration of 0.3 μmol/L) and the compound Ce6 (a final concentration of 100 μmol/L) were mixed, and exposed to LED white light (55 W) for 120 min; (B) The annealed TMV PQS5 solution without Ce6 was exposed to LED white light for 120 min; (C) The mixture of annealed TMV PQS5 and Ce6 was under the dark condition for 120 min. The samples covered with tin foils were placed at 4°C, and then detected by mass spectrometry. The separation was achieved with a Waters BEH C18 column (2.1 mm×100 mm, 1.7 μm) and column temperature was 60°C. Mobile phase A: water with 15 mM triethylamine (TFA) and 100 mM hexafluoroisopropanol (HFIP), mobile phase B: methanol-mobile phase A (50:50, v/v). Gradient started at 38% B, and reached 88% B at 7 min. 2.00 μL sample was used for each analysis with a flow rate of 0.20 mL/min. The parameters of ESI-MS in negative ion mode were as follows: capillary voltage, 2500 V; source temperature, 100°C; desolvation temperature, 250°C; cone gas flow, 50 L/h; desolvation gas flow 600L/h. Data acquisition and processing were performed with the UNIFI 1.9.4 software (Waters, Milford, MA USA) [37].

### 4.11 Anti-TMV activity of Ce6 upon photo-irradiation

TMV virions were inoculated on the whole tobacco leaves. After 3 h, the leaves were washed with distilled water and air-dried. Ce6 with different concentrations (50, 100, 200, and 500 μg/mL) were smeared on the whole leaves, and the same volume of solvent without Ce6 was used as control. For the photo-irradiation treatment of tobaccos, tobaccos were irradiated with the LED white light for 10 h, and then cultivated in the greenhouse with normal light irradiation for 14 h every day. The tobaccos under normal light were used as control. Three repetitions were conducted for each treatment. The local lesion numbers were recorded after TMV-inoculated for 4 days.

### 4.12 Phytotoxicity Evaluation

Phytotoxicity. *N*. *benthamiana* grew to 5–6 leaf stage was selected for phytotoxicity evaluation [38]. The compound solution (500 μg/mL) was sprayed on the leaves. The phenotypic symptoms were monitored visually during the experiment period.

Metabolic activity. The toxicity of chlorins was evaluated by analyzing its impact on the metabolic activity of BY-2 cells using resazurin reagent [24]. Non-fluorescent resazurin is taken up by viable cells and metabolized to fluorescent resorufin, which probably results from the action of several different redox enzymes in cell mitochondria, cytosol, and microsome. BY-2 cells (3-day after subcultures) were washed with 0.4 M mannitol buffer twice and collected by centrifugation at 700 rpm. The re-suspended cells were mixed with serially diluted compounds (0, 3.75, 6.25, 12.5, 25, 50, and 100 μM), and then maintained in the dark for 3 h at room temperature, followed by irradiation for 0.5 h. The metabolic activity of the cells with/without compounds was quantified directly by addition of resazurin solution (20 μg/mL) into the cell culture. Fluorescence measurements (excitation at 570 nm, emission at 590 nm) were performed after incubation for 3 h.

## Supporting information

S1 Fig 1H NMR spectra of TMV PQS5. (PDF)

S2 Fig smFRET analysis for the TMV PQS5. Schematic diagram of TMV PQS5d17 in the unfolded state (A) and the folded state (B). (C-E) Histogram fitting of the conformations in different solution with a multimodal Gaussian distribution. Each distribution is derived from more than 150 individual curves. Experiments were performed at room temperature without/with potassium (C, D) in 10 mM Tris-HCl buffer (pH 7.4), supplemented with 3 μM NMM (E). (F) Proportion of high FRET species in Fig 2C–2E. (G-H) Representative FRET traces of TMV PQS5d17 in the folded state and in the unfolded state.
(PDF)

S3 Fig Intensity fluctuations of individual TMV PQS5d17. The fluorescent molecules were continuously excited at a low laser intensity, and then the laser intensity was suddenly increased and maintained at this level until a photobleaching occurs. Representative traces of cy3 (A) and cy5 (B) fluorescence intensity changed with time, which indicated the presence of only one fluorophore in TMV PQS5d17. Experiments were performed at room temperature in 10 mM Tris-HCl buffer (pH 7.4) with 100 mM KCl.
(PDF)

S4 Fig CD spectra of TMV-PQS5 in solution with KCl at different concentrations. (PDF)

S5 Fig Map of the Ph7lic14-TMV U1-GPF plasmid bearing TMV PQS5. (PDF)

S6 Fig Structures of synthesized and natural G-quadruplex ligands. (PDF)

S7 Fig Anti-TMV activities of compounds at different concentrations in N. benthamiana plants using digital fluorescence visual screening. The green fluorescence signal represented the viral replication in plants that were inoculated with TMV−GFP construct at different hours post inoculation (hpi).
(PDF)

S8 Fig Evaluation of the interaction between TMV PQS5 and two ligands. (A) Ultraviolet-visible absorption spectra of TMPyP4 (5μM) with addition of TMV PQS5 (10 μM). (B) Fluorescence emission spectra of 5 μM TMPyP4 in the presence of gradient RNA G-quadruplex of TMV PQS5 (0 μM, 1 μM, 2 μM, 4 μM, 6 μM, 8 μM and 10 μM), λex = 440 nm. Fluorescence emission of compound TMPyP4 alone is shown in black. (C) Ultraviolet-visible absorption spectra of BRACO-19 (5μM) with addition of TMV PQS5 (10 μM). (D) Fluorescence emission spectra of 5 μM BRACO-19 in the presence of gradient RNA G-quadruplex of TMV PQS5 (0 μM, 1 μM, 2 μM, 4 μM, 6 μM and 8 μM), λex = 371 nm. Fluorescence emission of compound BRACO-19 alone is shown in black.
(PDF)

S9 Fig Anti-TMV activities of G-quadruplex ligands (Braco-19, TMPyP4) in N. benthamiana plants. The green fluorescence signal represented the viral replication in plants that were inoculated with TMV−GFP construct at different hours post inoculation (hpi).
(PDF)

S10 Fig Evaluation of the interaction between TMV PQS5 and two plant isoquinoline alkaloids. (A) Ultraviolet-visible absorption spectra of Sanguinarine (20 μM) with addition of RNA G-quadruplex TMV PQS5 (10μM). (B) Fluorescence emission spectra of 20 μM Sanguinarine in the presence of RNA G-quadruplex TMV PQS5 (10 μM), λex = 334 nm. (C) Ultraviolet-visible absorption spectra of Tryptanthrin (20 μM) with addition of RNA G-quadruplex TMV PQS5 (10 μM). (D) Fluorescence emission spectra of 20 μM Tryptanthrin in the presence of RNA G-quadruplex TMV PQS5 (10 μM), λex = 338 nm. Fluorescence emission of compounds alone is shown in black.
(PDF)

S11 Fig Representative photosensitizers used in this study. (A) porphyrins, (B) chlorins, (C) acridines, and (D) RB.
(PDF)

S12 Fig UV spectra of TMV PQS5 (1 μmol/L) with or without different photosensitive compounds (1 μmol/L). (PDF)

S13 Fig CD spectra of 15 μmol/L TMV PQS5 with 80 μmol/L Ce6. (PDF)

S14 Fig Mechanism of detecting ROS by DCFH-DA. (PDF)

S15 Fig Fluorescence images for ROS emitted by Ce6 in BY-2 cells upon photo-irradiation. Upper: BY-2 cells without Ce6. Bottom: BY-2 cells treated with 10 μM Ce6 for 2 h. DIC images of cells and fluorescent images of DCFH-DA are presented. Excitation wavelength, 488 nm. Scale bar, 20 μm.
(PDF)

S16 Fig NAC decreases the ROS level produced by Ce6. Fluorescence spectra of 10 μM DCFH-DA in the presence of 10 μM Ce6 (B) with or without (A) 10 mM NAC upon photo-irradiation. The spectra at irradiation time points 0 and 120 min are highlighted in black and red, respectively. (C) Representative fluorescence images of living BY-2 cells after photo-irradiation. Upper: BY-2 cells treated with 10 μM Ce6. Middle: BY-2 cells treated with 10 μM Ce6 and 10 mM NAC. Bottom: BY-2 cells without Ce6 nor NAC served as mock. DIC images of cells and fluorescent images of DCFH-DA are presented. λex = 488 nm, λem = 523 nm. Scale bar, 20 μm.
(PDF)

S17 Fig 20% denaturing polyacrylamide gel electrophoresis of 0.3 μM TMV PQS5 after photo-irradiation for the indicated periods at 25°C. (A) Ce6 mixing with PQS5 under strong LED light conditions for different time (0, 15, 30, 60, 90, 120 min). (B) 0.3 μM PQS5 without Ce6 under strong LED light conditions for different time (0,120 min). D120 represents the sample treated at dark for 120 min.
(PDF)

S18 Fig Mass spectra of TMV PQS5 in the presence of Ce6 under the LED light condition or dark condition. The treated procedures for sample A-C were as follows: (A) The mixture of the annealed TMV PQS5 (0.3 μmol/L) and compound Ce6 (100 μmol/L) with the exposion of the LED white light (55 W) for 120 min; (B) The annealed TMV PQS5 solution without Ce6 under the LED white light for 120 min; (C) The mixture of annealed TMV PQS5 and Ce6 under the dark condition for 120 min.
(PDF)

S19 Fig Phytotoxicity evaluation of chlorins on Nicotiana benthamiana. After 5 μM Ce6 or Ce6TME was sprayed on Nicotiana benthamiana for 7 days, the growth of tobacco was examined. This indicated that Ce6 or Ce6TME did not affect the growth and development of tobacco.
(PDF)

S20 Fig Metabolic activities of BY-2 cells treated with Ce6 at different concentrations under light or dark. Fluorescent changes of resorufin were used to reflect the metabolic activities.
(PDF)

S1 Table Reference genome sequences of plant viruses from 35 classified families. (PDF)

S2 Table Pattern conservation, the sequence conservation, and the folding potential of PQSs in TMV genomes. (PDF)

S3 Table Sequences of oligomers used in RNA stop assay. (PDF)

S4 Table List of primers for reverse transcription and real time RT-PCR. (PDF)

S5 Table Antiviral activities of NMM against TMV in vivo. (PDF)

S6 Table Information of primers used in plasmid construction. (PDF)

S7 Table Anti-TMV activities of different photosensitive compounds in vivo. (PDF)
