# Supplementary figures and images for: G-quadruplex in the TMV Genome Regulates Viral Proliferation and Acts as Antiviral Target of Photodynamic Therapy
Source: PLoS Pathog. 2023 Dec 7;19(12):e1011796. doi: 10.1371/journal.ppat.1011796 (PMC10760922; doi:10.1371/journal.ppat.1011796)

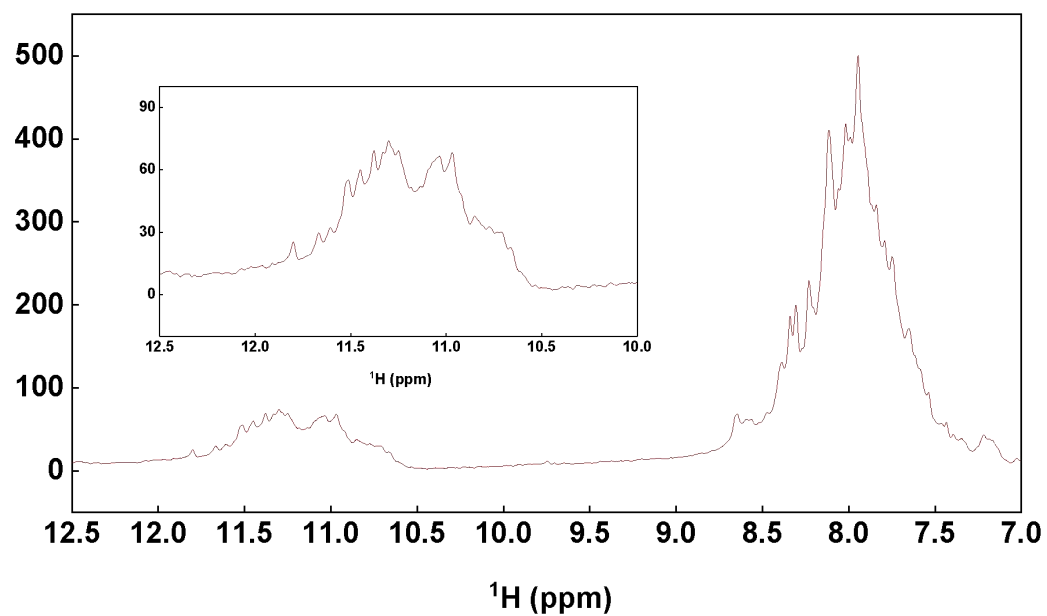

**Fig S1.  $^1\text{H}$  NMR spectra of TMV PQS5.**

Supplement: S1 Fig — (PDF) [file ppat.1011796.s001.pdf]

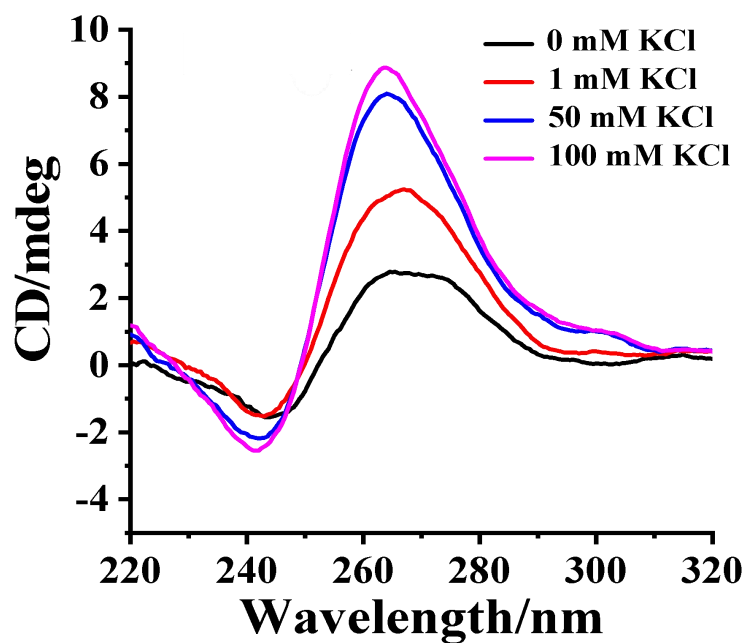

**Fig S4. CD spectra of TMV-PQS5 in solution with KCl at different concentrations.**

Supplement: S4 Fig — (PDF) [file ppat.1011796.s004.pdf]

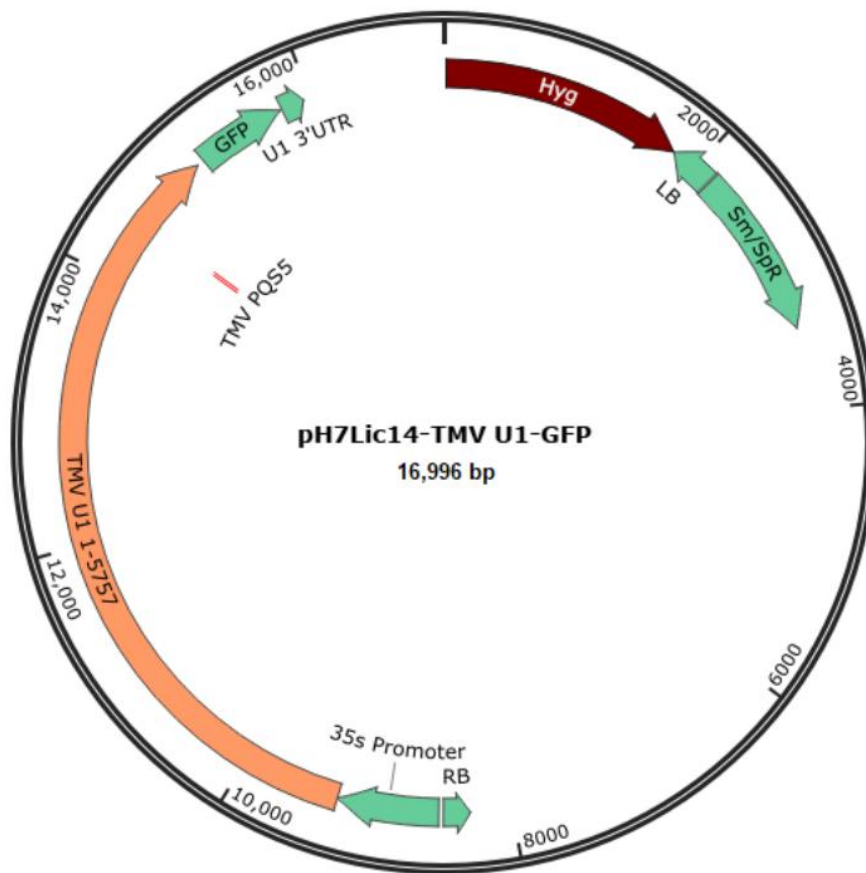

**Fig S5. Map of the Ph7lic14-TMV U1-GPF plasmid bearing TMV PQS5.**

Supplement: S5 Fig — (PDF) [file ppat.1011796.s005.pdf]

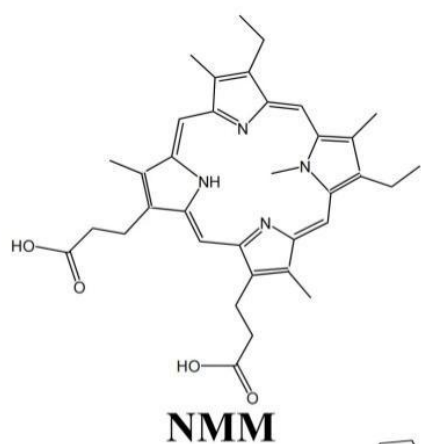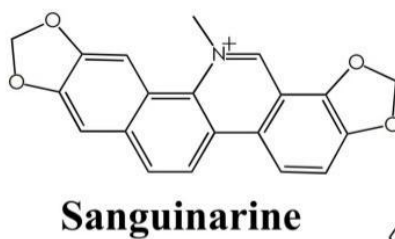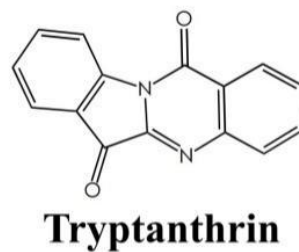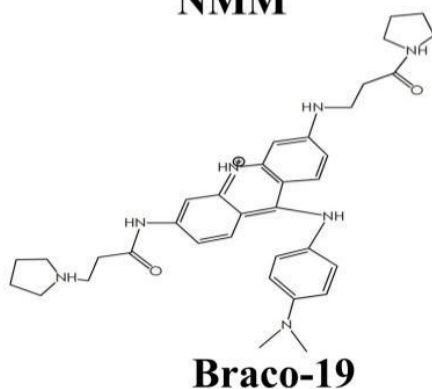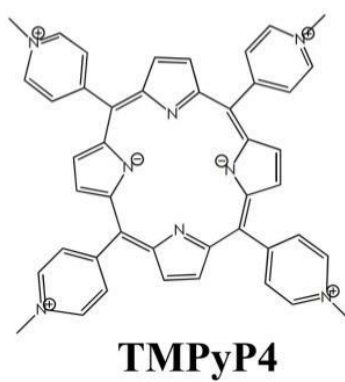

**Fig S6. Structures of synthesized and natural G-quadruplex ligands.**

Supplement: S6 Fig — (PDF) [file ppat.1011796.s006.pdf]

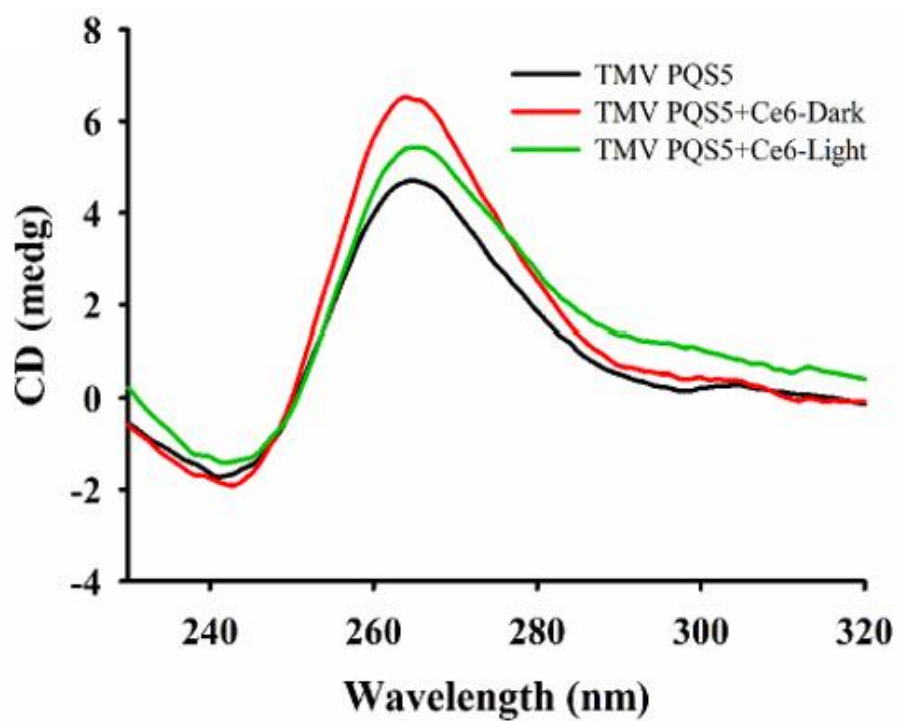

**Fig S13. CD spectra of 15  $\mu\text{mol/L}$  TMV PQS5 with 80  $\mu\text{mol/L}$  Ce6.**

Supplement: S13 Fig — (PDF) [file ppat.1011796.s013.pdf]

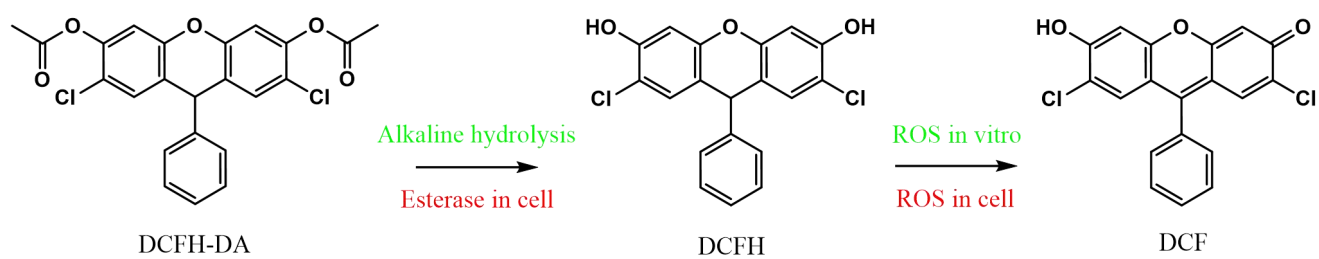

**Fig S14. Mechanism of detecting ROS by DCFH-DA.**

Supplement: S14 Fig — (PDF) [file ppat.1011796.s014.pdf]
