# Supplementary material for: G-quadruplex in the TMV Genome Regulates Viral Proliferation and Acts as Antiviral Target of Photodynamic Therapy
Source: PLoS Pathog. 2023 Dec 7;19(12):e1011796. doi: 10.1371/journal.ppat.1011796 (PMC10760922; doi:10.1371/journal.ppat.1011796)
Supplement: S2 Fig — Schematic diagram of TMV PQS5d17 in the unfolded state (A) and the folded state (B). (C-E) Histogram fitting of the conformations in different solution with a multimodal Gaussian distribution. Each distribution is derived from more than 150 individual curves. Experiments were performed at room temperature without/with potassium (C, D) in 10 mM Tris-HCl buffer (pH 7.4), supplemented with 3 μM NMM (E). (F) Proportion of high FRET species in Fig 2C–2E. (G-H) Representative FRET traces of TMV PQS5d17 in the folded state and in the unfolded state. (PDF) [file ppat.1011796.s002.pdf]

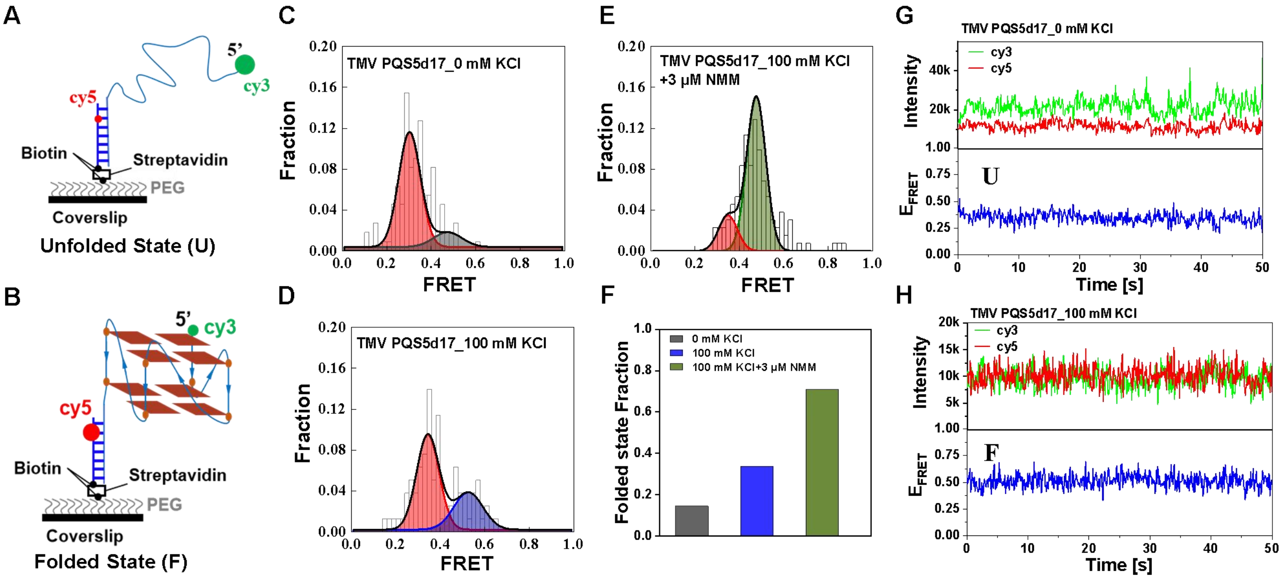

Fig S2. smFRET analysis for the TMV PQS5. Schematic diagram of TMV PQS5d17 in the unfolded state (A) and the folded state (B). (C-E) Histogram fitting of the conformations in different solution with a multimodal Gaussian distribution. Each distribution is derived from more than 150 individual curves. Experiments were performed at room temperature without/with potassium (C, D) in 10 mM Tris-HCl buffer (pH 7.4), supplemented with 3 μM NMM (E). (F) Proportion of high FRET species in Figure R2C-2E. (G-H) Representative FRET traces of TMV PQS5d17 in the folded state and in the unfolded state.
