# Supplementary material for: G-quadruplex in the TMV Genome Regulates Viral Proliferation and Acts as Antiviral Target of Photodynamic Therapy
Source: PLoS Pathog. 2023 Dec 7;19(12):e1011796. doi: 10.1371/journal.ppat.1011796 (PMC10760922; doi:10.1371/journal.ppat.1011796)
Supplement: S3 Fig — The fluorescent molecules were continuously excited at a low laser intensity, and then the laser intensity was suddenly increased and maintained at this level until a photobleaching occurs. Representative traces of cy3 (A) and cy5 (B) fluorescence intensity changed with time, which indicated the presence of only one fluorophore in TMV PQS5d17. Experiments were performed at room temperature in 10 mM Tris-HCl buffer (pH 7.4) with 100 mM KCl. (PDF) [file ppat.1011796.s003.pdf]

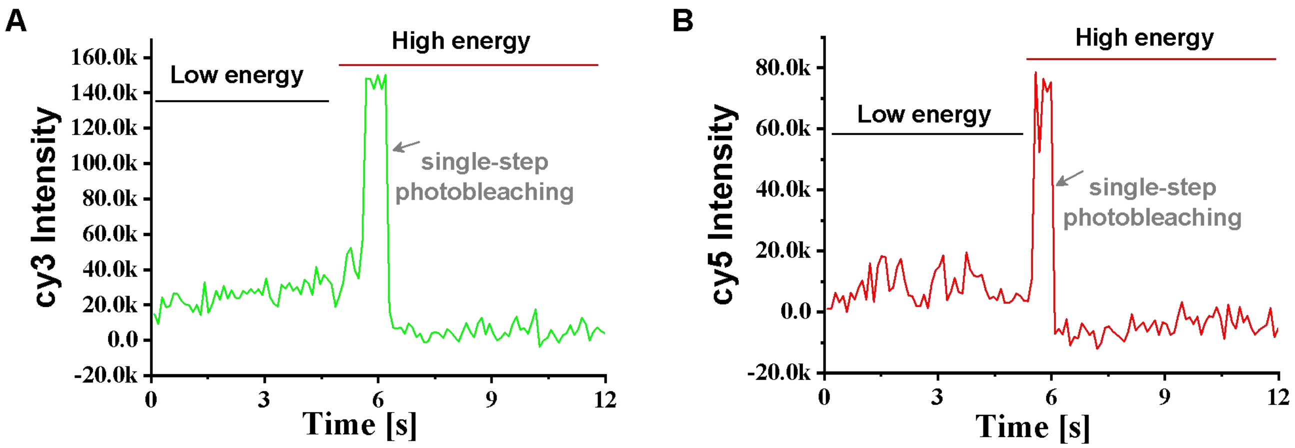

Fig S3. Intensity fluctuations of individual TMV PQS5d17. The fluorescent molecules were continuously excited at a low laser intensity, and then the laser intensity was suddenly increased and maintained at this level until a photobleaching occurs. Representative traces of cy3 (A) and cy5 (B) fluorescence intensity changed with time, which indicated the presence of only one fluorophore in TMV PQS5d17. Experiments were performed at room temperature in 10 mM Tris-HCl buffer (pH 7.4) with 100 mM KCl.
