# Supplementary material for: G-quadruplex in the TMV Genome Regulates Viral Proliferation and Acts as Antiviral Target of Photodynamic Therapy
Source: PLoS Pathog. 2023 Dec 7;19(12):e1011796. doi: 10.1371/journal.ppat.1011796 (PMC10760922; doi:10.1371/journal.ppat.1011796)
Supplement: S7 Fig — The green fluorescence signal represented the viral replication in plants that were inoculated with TMV−GFP construct at different hours post inoculation (hpi). (PDF) [file ppat.1011796.s007.pdf]

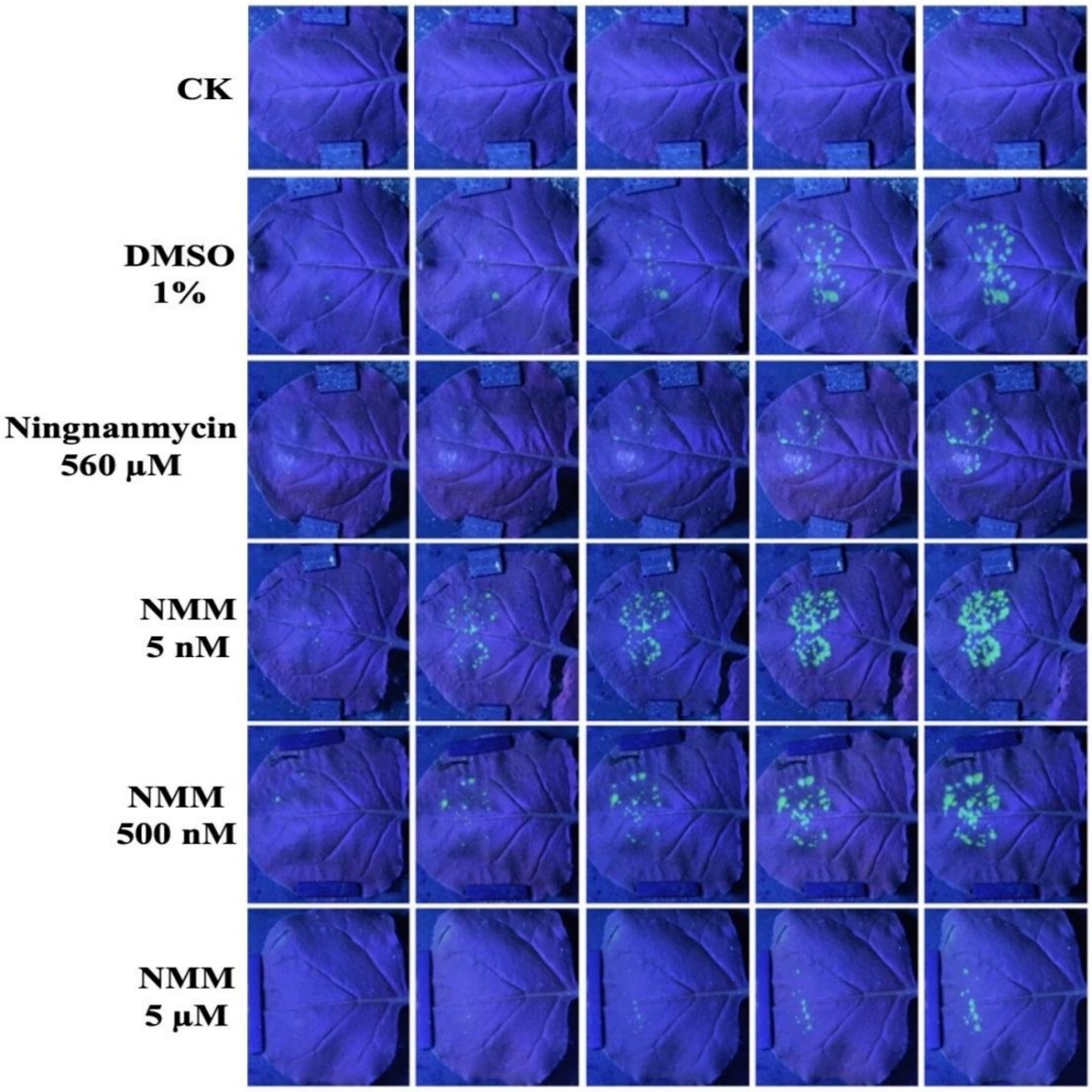

Fig S7. Anti-TMV activities of compounds at different concentrations in *N. benthamiana* plants using digital fluorescence visual screening. The green fluorescence signal represented the viral replication in plants that were inoculated with TMV−GFP construct at different hours post inoculation (hpi).
