# Supplementary material for: G-quadruplex in the TMV Genome Regulates Viral Proliferation and Acts as Antiviral Target of Photodynamic Therapy
Source: PLoS Pathog. 2023 Dec 7;19(12):e1011796. doi: 10.1371/journal.ppat.1011796 (PMC10760922; doi:10.1371/journal.ppat.1011796)
Supplement: S8 Fig — (A) Ultraviolet-visible absorption spectra of TMPyP4 (5μM) with addition of TMV PQS5 (10 μM). (B) Fluorescence emission spectra of 5 μM TMPyP4 in the presence of gradient RNA G-quadruplex of TMV PQS5 (0 μM, 1 μM, 2 μM, 4 μM, 6 μM, 8 μM and 10 μM), λex = 440 nm. Fluorescence emission of compound TMPyP4 alone is shown in black. (C) Ultraviolet-visible absorption spectra of BRACO-19 (5μM) with addition of TMV PQS5 (10 μM). (D) Fluorescence emission spectra of 5 μM BRACO-19 in the presence of gradient RNA G-quadruplex of TMV PQS5 (0 μM, 1 μM, 2 μM, 4 μM, 6 μM and 8 μM), λex = 371 nm. Fluorescence emission of compound BRACO-19 alone is shown in black. (PDF) [file ppat.1011796.s008.pdf]

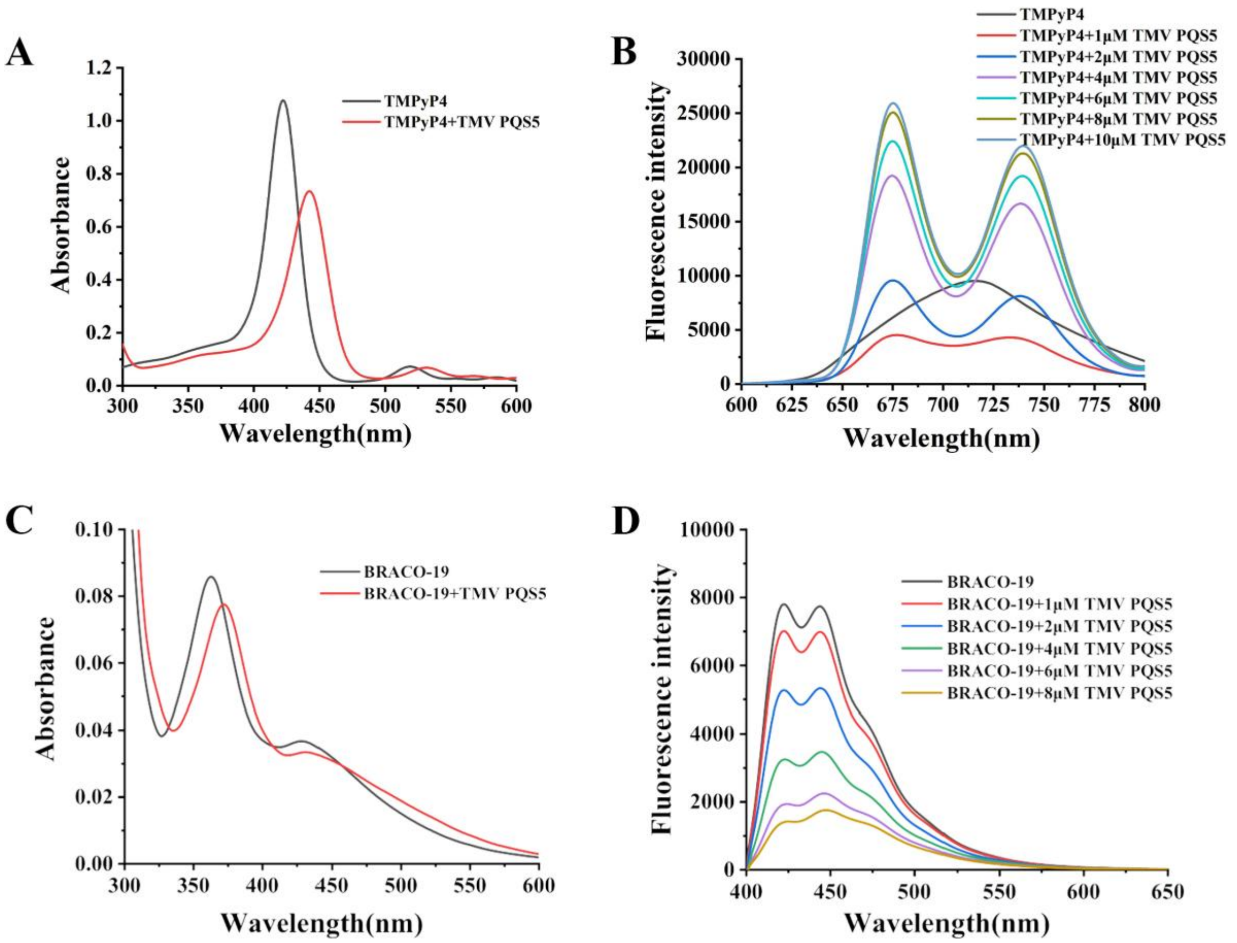

Fig S8. Evaluation of the interaction between TMV PQS5 and two ligands. (A) Ultraviolet-visible absorption spectra of TMPyP4 (5μM) with addition of TMV PQS5 (10 μM). (B) Fluorescence emission spectra of 5 μM TMPyP4 in the presence of gradient RNA G-quadruplex of TMV PQS5 (0 μM, 1 μM, 2 μM, 4 μM, 6 μM, 8 μM and 10 μM), λ_{ex} =440 nm. Fluorescence emission of compound TMPyP4 alone is shown in black. (C) Ultraviolet-visible absorption spectra of BRACO-19 (5μM) with addition of TMV PQS5 (10 μM). (D) Fluorescence emission spectra of 5 μM BRACO-19 in the presence of gradient RNA G-quadruplex of TMV PQS5 (0 μM, 1 μM, 2 μM, 4 μM, 6 μM and 8 μM), λ_{ex} =371 nm. Fluorescence emission of compound BRACO-19 alone is shown in black.
