# Supplementary material for: G-quadruplex in the TMV Genome Regulates Viral Proliferation and Acts as Antiviral Target of Photodynamic Therapy
Source: PLoS Pathog. 2023 Dec 7;19(12):e1011796. doi: 10.1371/journal.ppat.1011796 (PMC10760922; doi:10.1371/journal.ppat.1011796)
Supplement: S10 Fig — (A) Ultraviolet-visible absorption spectra of Sanguinarine (20 μM) with addition of RNA G-quadruplex TMV PQS5 (10μM). (B) Fluorescence emission spectra of 20 μM Sanguinarine in the presence of RNA G-quadruplex TMV PQS5 (10 μM), λex = 334 nm. (C) Ultraviolet-visible absorption spectra of Tryptanthrin (20 μM) with addition of RNA G-quadruplex TMV PQS5 (10 μM). (D) Fluorescence emission spectra of 20 μM Tryptanthrin in the presence of RNA G-quadruplex TMV PQS5 (10 μM), λex = 338 nm. Fluorescence emission of compounds alone is shown in black. (PDF) [file ppat.1011796.s010.pdf]

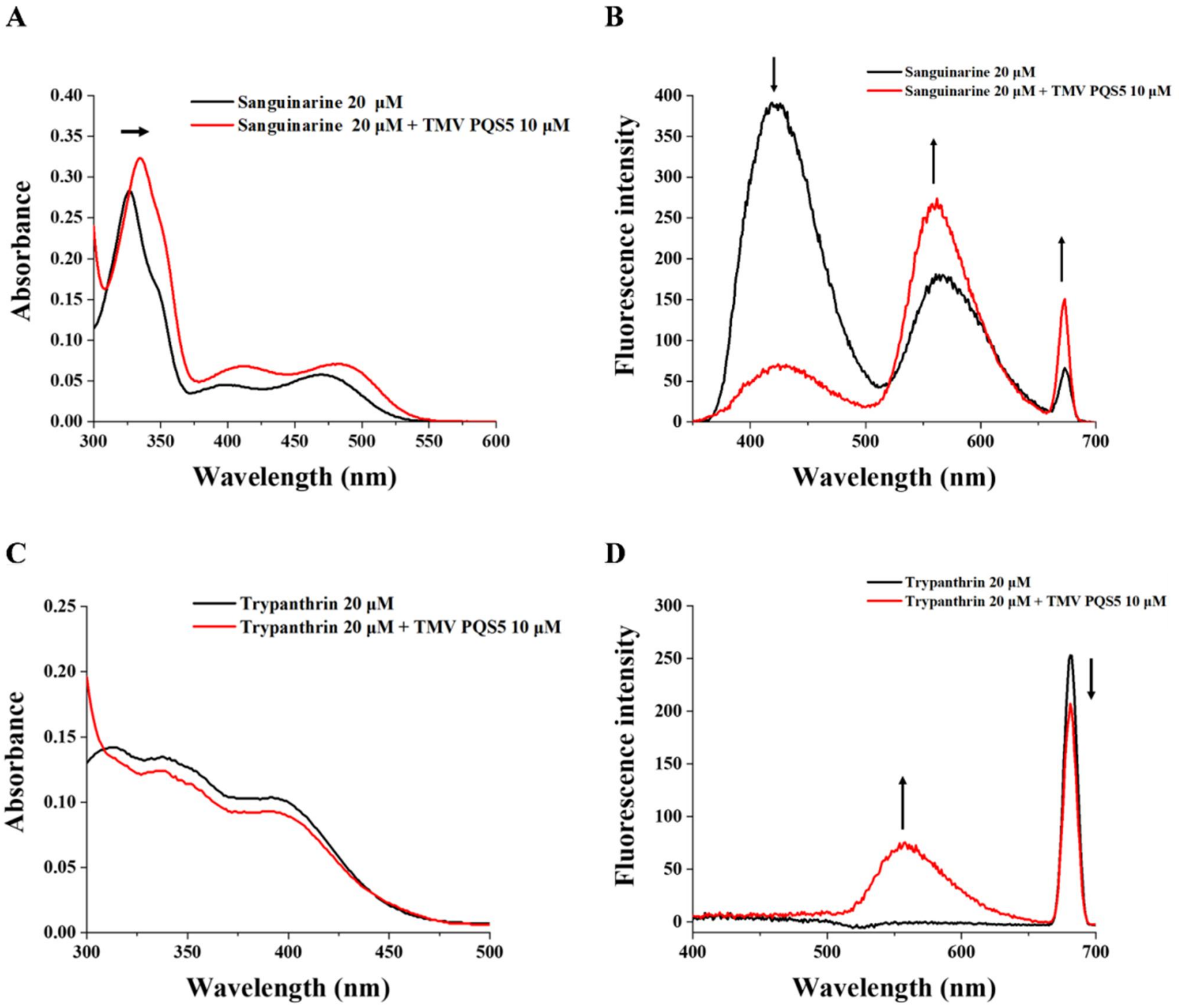

Fig S10. Evaluation of the interaction between TMV PQS5 and two plant isoquinoline alkaloids. (A) Ultraviolet-visible absorption spectra of Sanguinarine (20 μM) with addition of RNA G-quadruplex TMV PQS5 (10μM). (B) Fluorescence emission spectra of 20 μM Sanguinarine in the presence of RNA G-quadruplex TMV PQS5 (10 μM), λ_{ex} =334 nm. (C) Ultraviolet-visible absorption spectra of Tryptanthrin (20 μM) with addition of RNA G-quadruplex TMV PQS5 (10 μM). (D) Fluorescence emission spectra of 20 μM Tryptanthrin in the presence of RNA G-quadruplex TMV PQS5 (10 μM), λ_{ex} =338 nm. Fluorescence emission of compounds alone is shown in black.
