# Supplementary material for: G-quadruplex in the TMV Genome Regulates Viral Proliferation and Acts as Antiviral Target of Photodynamic Therapy
Source: PLoS Pathog. 2023 Dec 7;19(12):e1011796. doi: 10.1371/journal.ppat.1011796 (PMC10760922; doi:10.1371/journal.ppat.1011796)
Supplement: S11 Fig — (A) porphyrins, (B) chlorins, (C) acridines, and (D) RB. (PDF) [file ppat.1011796.s011.pdf]

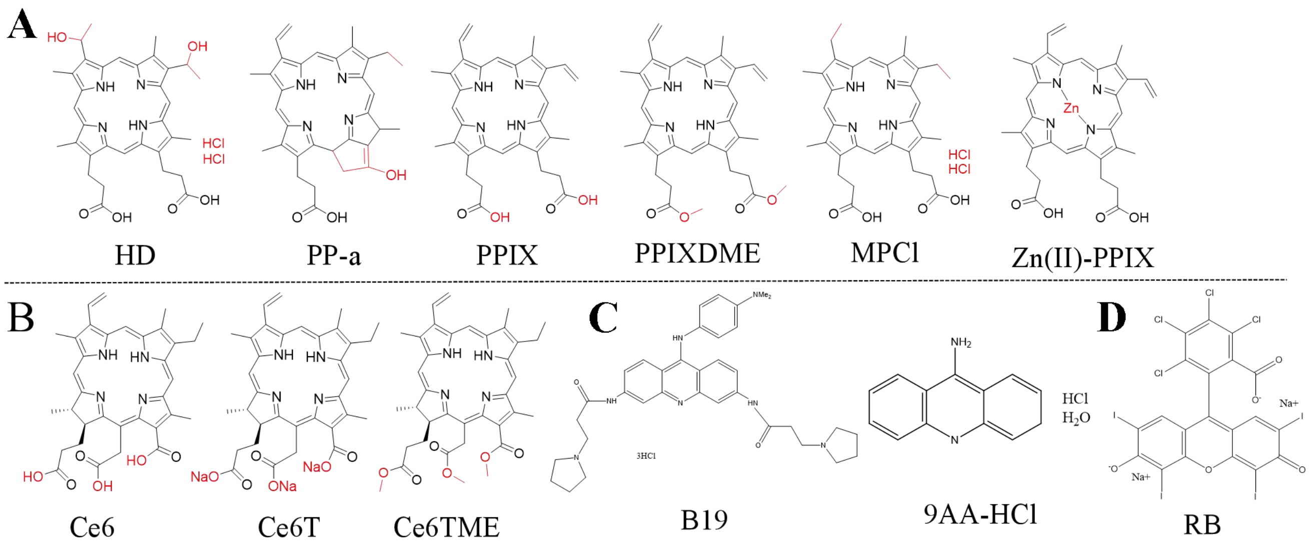

Fig S11. Representative photosensitizers used in this study.

(A) porphyrins, (B) chlorins, (C) acridines, and (D) RB.
