# Supplementary material for: G-quadruplex in the TMV Genome Regulates Viral Proliferation and Acts as Antiviral Target of Photodynamic Therapy
Source: PLoS Pathog. 2023 Dec 7;19(12):e1011796. doi: 10.1371/journal.ppat.1011796 (PMC10760922; doi:10.1371/journal.ppat.1011796)
Supplement: S12 Fig — (PDF) [file ppat.1011796.s012.pdf]

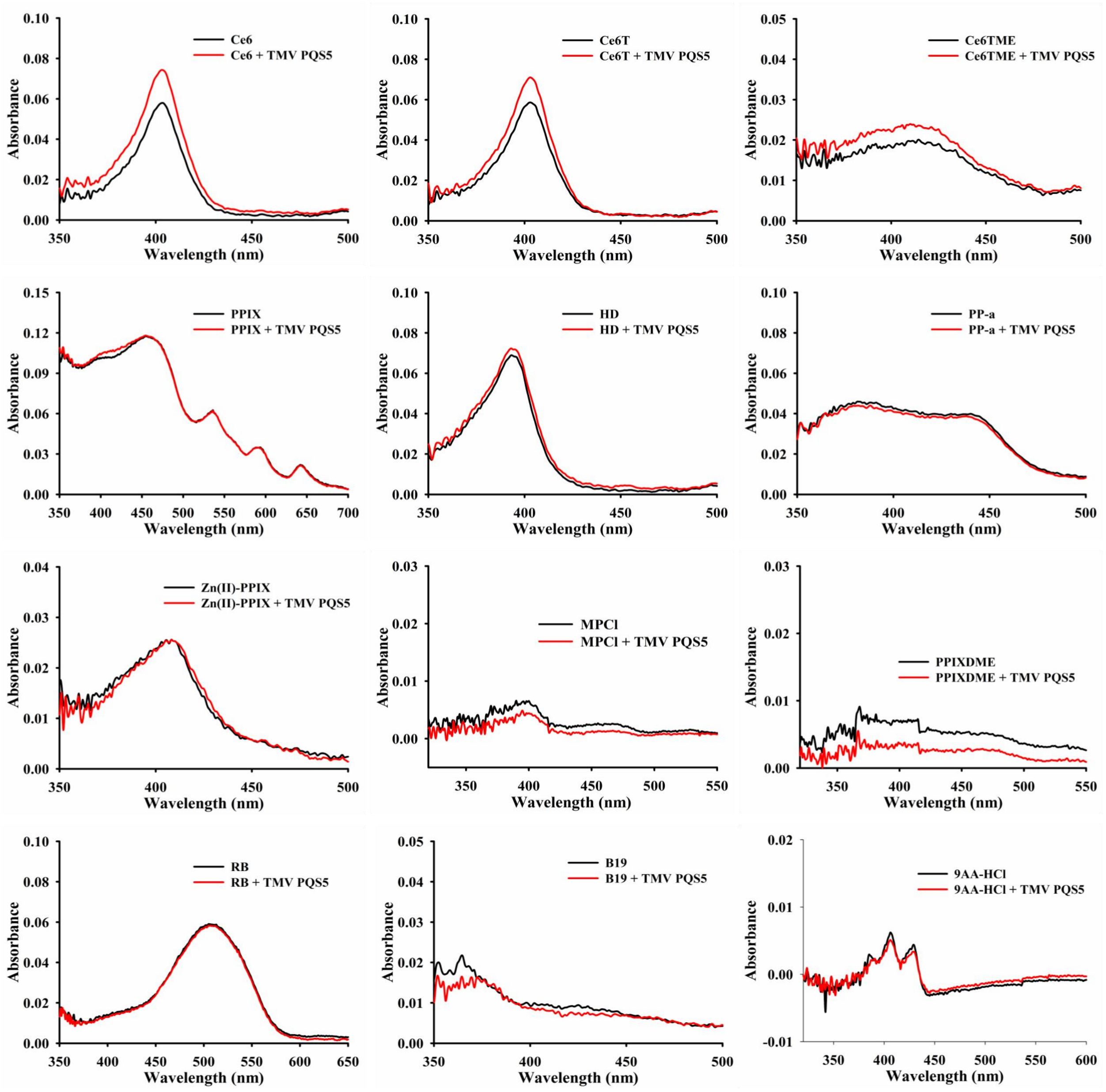

Fig S12. UV spectra of TMV PQS5 (1 μmol/L) with or without different photosensitive compounds (1 μmol/L).
