# Supplementary material for: G-quadruplex in the TMV Genome Regulates Viral Proliferation and Acts as Antiviral Target of Photodynamic Therapy
Source: PLoS Pathog. 2023 Dec 7;19(12):e1011796. doi: 10.1371/journal.ppat.1011796 (PMC10760922; doi:10.1371/journal.ppat.1011796)
Supplement: S15 Fig — Upper: BY-2 cells without Ce6. Bottom: BY-2 cells treated with 10 μM Ce6 for 2 h. DIC images of cells and fluorescent images of DCFH-DA are presented. Excitation wavelength, 488 nm. Scale bar, 20 μm. (PDF) [file ppat.1011796.s015.pdf]

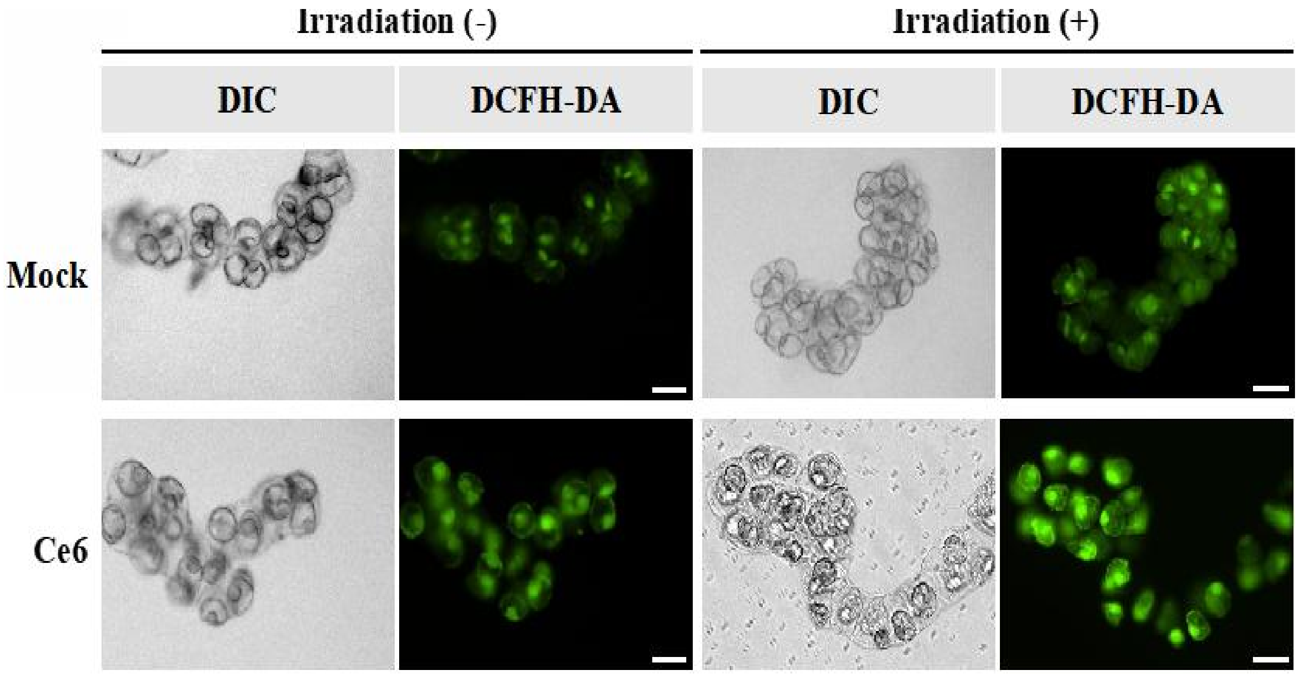

Fig S15. Fluorescence images for ROS emitted by Ce6 in BY-2 cells upon photo-irradiation. Upper: BY-2 cells without Ce6. Bottom: BY-2 cells treated with 10 μM Ce6 for 2 h. DIC images of cells and fluorescent images of DCFH-DA are presented. Excitation wavelength, 488 nm. Scale bar, 20 μm.
