# Supplementary material for: G-quadruplex in the TMV Genome Regulates Viral Proliferation and Acts as Antiviral Target of Photodynamic Therapy
Source: PLoS Pathog. 2023 Dec 7;19(12):e1011796. doi: 10.1371/journal.ppat.1011796 (PMC10760922; doi:10.1371/journal.ppat.1011796)
Supplement: S16 Fig — Fluorescence spectra of 10 μM DCFH-DA in the presence of 10 μM Ce6 (B) with or without (A) 10 mM NAC upon photo-irradiation. The spectra at irradiation time points 0 and 120 min are highlighted in black and red, respectively. (C) Representative fluorescence images of living BY-2 cells after photo-irradiation. Upper: BY-2 cells treated with 10 μM Ce6. Middle: BY-2 cells treated with 10 μM Ce6 and 10 mM NAC. Bottom: BY-2 cells without Ce6 nor NAC served as mock. DIC images of cells and fluorescent images of DCFH-DA are presented. λex = 488 nm, λem = 523 nm. Scale bar, 20 μm. (PDF) [file ppat.1011796.s016.pdf]

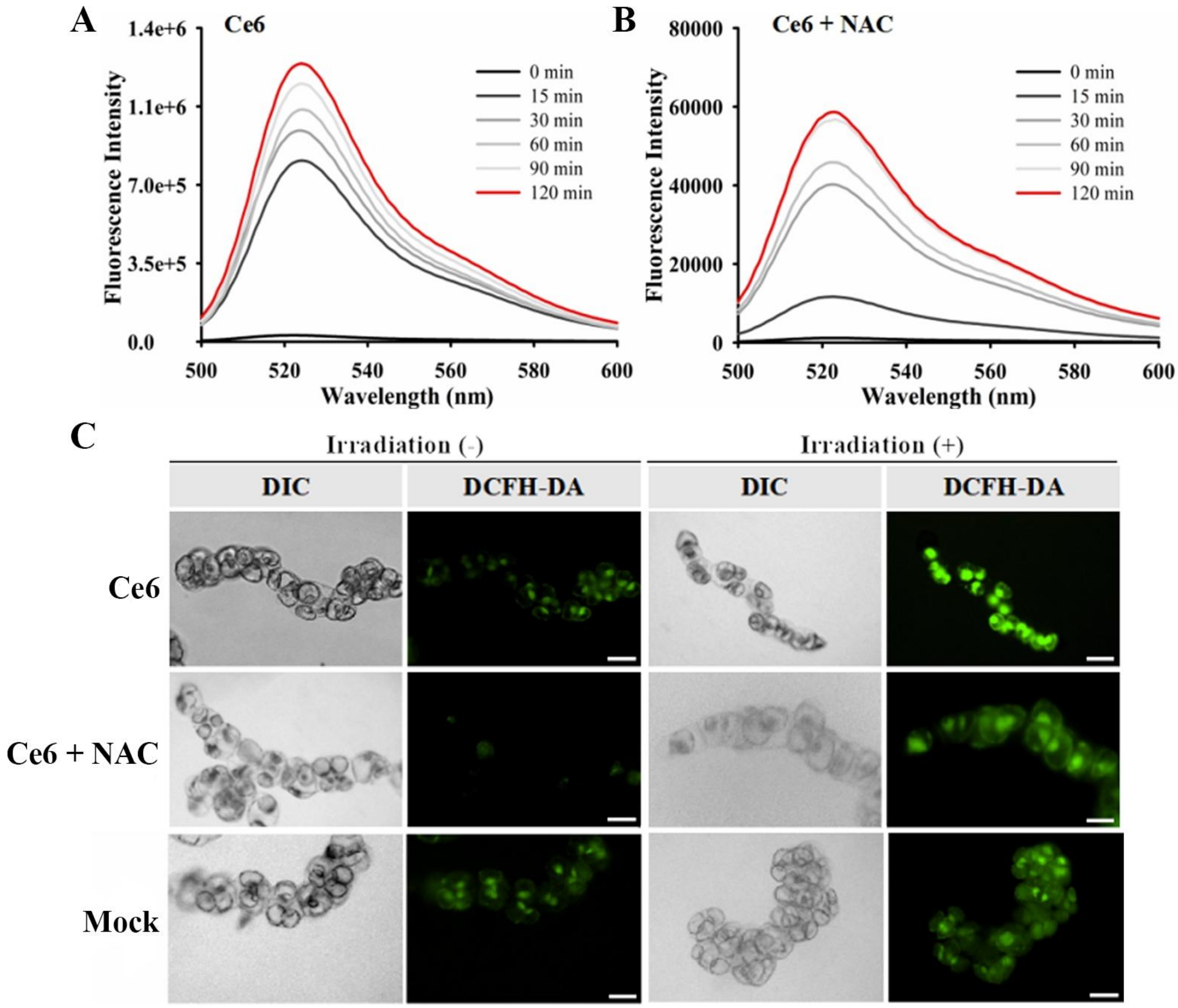

Fig S16. NAC decreases the ROS level produced by Ce6. Fluorescence spectra of 10 μM DCFH-DA in the presence of 10 μM Ce6 (B) with or without (A) 10 mM NAC upon photo-irradiation. The spectra at irradiation time points 0 and 120 min are highlighted in black and red, respectively. (C) Representative fluorescence images of living BY-2 cells after photo-irradiation. Upper: BY-2 cells treated with 10 μM Ce6. Middle: BY-2 cells treated with 10 μM Ce6 and 10 mM NAC. Bottom: BY-2 cells without Ce6 nor NAC served as mock. DIC images of cells and fluorescent images of DCFH-DA are presented. λ_{ex} =488 nm, λ_{em} =523 nm. Scale bar, 20 μm.
