# Supplementary material for: G-quadruplex in the TMV Genome Regulates Viral Proliferation and Acts as Antiviral Target of Photodynamic Therapy
Source: PLoS Pathog. 2023 Dec 7;19(12):e1011796. doi: 10.1371/journal.ppat.1011796 (PMC10760922; doi:10.1371/journal.ppat.1011796)
Supplement: S17 Fig — (A) Ce6 mixing with PQS5 under strong LED light conditions for different time (0, 15, 30, 60, 90, 120 min). (B) 0.3 μM PQS5 without Ce6 under strong LED light conditions for different time (0,120 min). D120 represents the sample treated at dark for 120 min. (PDF) [file ppat.1011796.s017.pdf]

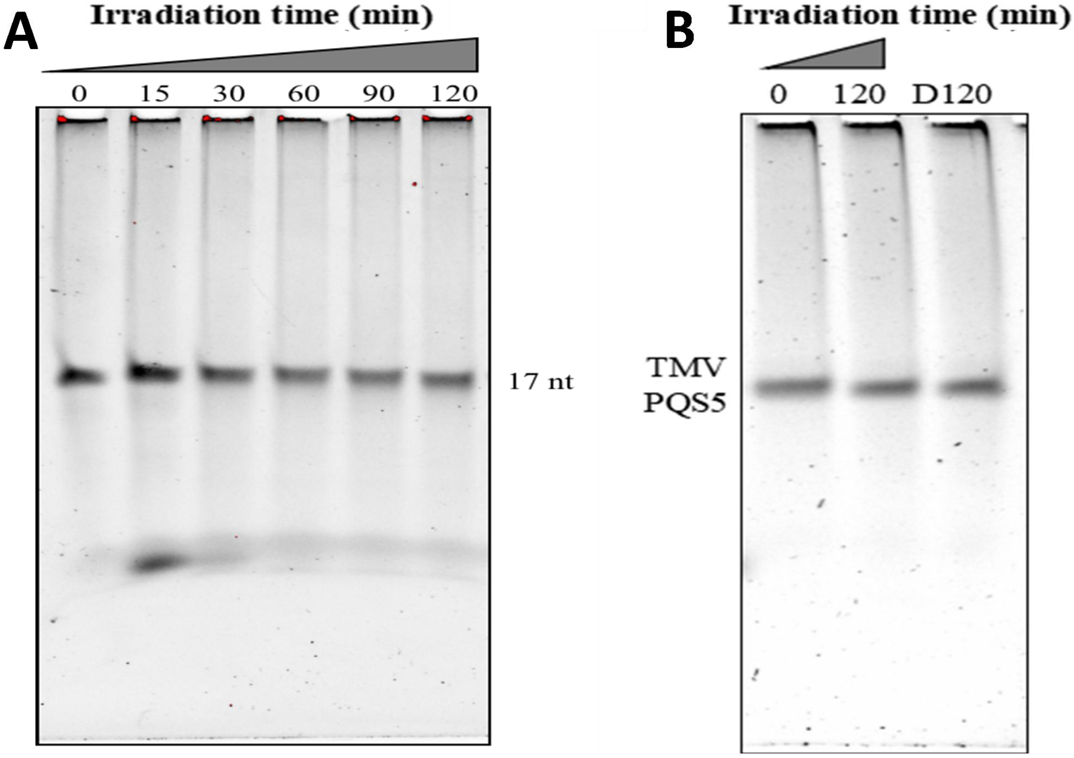

Fig S17. 20% denaturing polyacrylamide gel electrophoresis of 0.3 μM TMV PQS5 after photo-irradiation for the indicated periods at 25 °C. (A) Ce6 mixing with PQS5 under strong LED light conditions for different time (0, 15, 30, 60, 90, 120 min). (B) 0.3 μM PQS5 without Ce6 under strong LED light conditions for different time (0,120 min). D120 represents the sample treated at dark for 120 min.
