# Supplementary material for: G-quadruplex in the TMV Genome Regulates Viral Proliferation and Acts as Antiviral Target of Photodynamic Therapy
Source: PLoS Pathog. 2023 Dec 7;19(12):e1011796. doi: 10.1371/journal.ppat.1011796 (PMC10760922; doi:10.1371/journal.ppat.1011796)
Supplement: S18 Fig — The treated procedures for sample A-C were as follows: (A) The mixture of the annealed TMV PQS5 (0.3 μmol/L) and compound Ce6 (100 μmol/L) with the exposion of the LED white light (55 W) for 120 min; (B) The annealed TMV PQS5 solution without Ce6 under the LED white light for 120 min; (C) The mixture of annealed TMV PQS5 and Ce6 under the dark condition for 120 min. (PDF) [file ppat.1011796.s018.pdf]

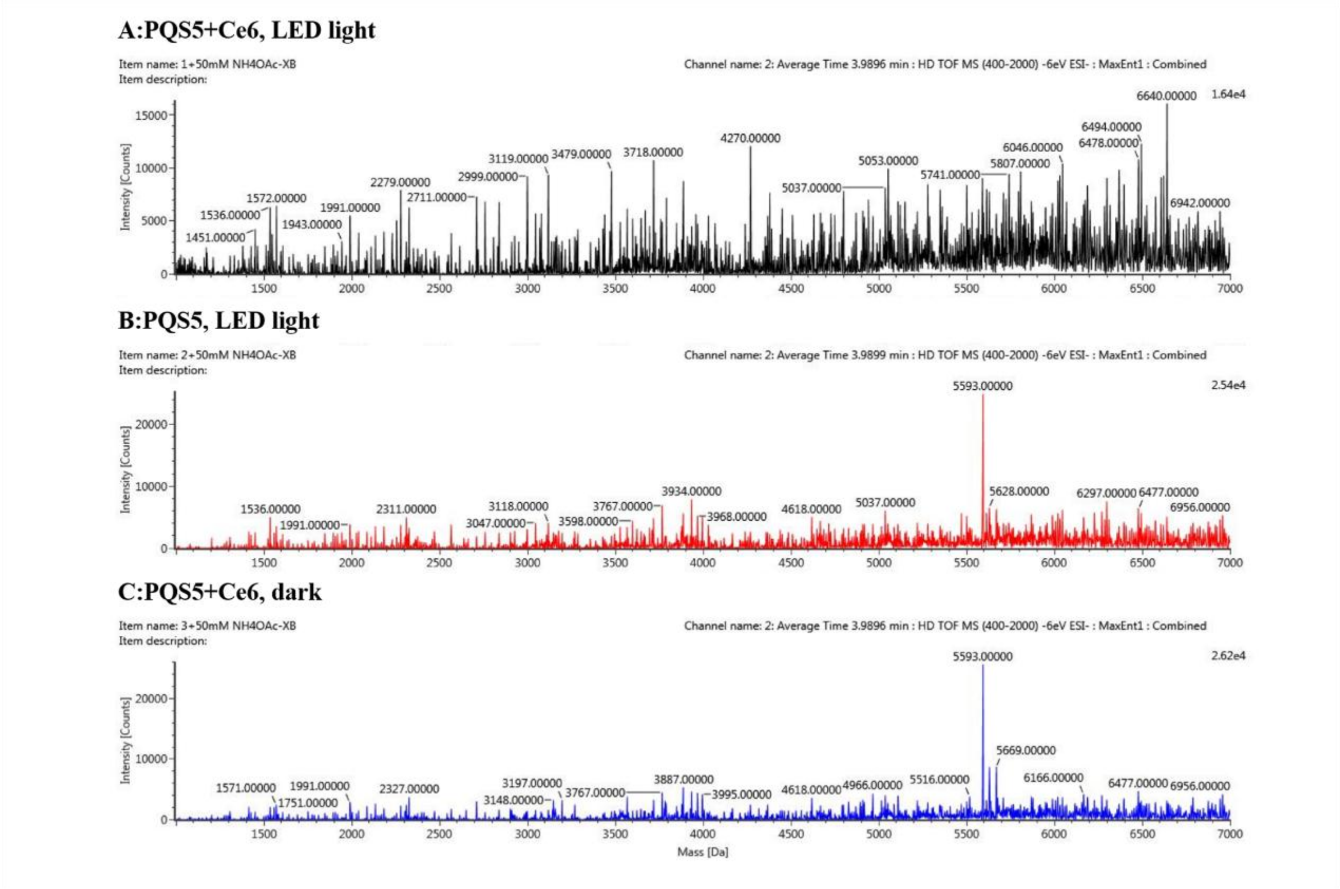

Fig S18. Mass spectra of TMV PQS5 in the presence of Ce6 under the LED light condition or dark condition. The treated procedures for sample A-C were as follows: (A) The mixture of the annealed TMV PQS5 (0.3 μmol/L) and compound Ce6 (100 μmol/L) with the exposion of the LED white light (55 W) for 120 min; (B) The annealed TMV PQS5 solution without Ce6 under the LED white light for 120 min; (C) The mixture of annealed TMV PQS5 and Ce6 under the dark condition for 120 min.
