# Supplementary material for: G-quadruplex in the TMV Genome Regulates Viral Proliferation and Acts as Antiviral Target of Photodynamic Therapy
Source: PLoS Pathog. 2023 Dec 7;19(12):e1011796. doi: 10.1371/journal.ppat.1011796 (PMC10760922; doi:10.1371/journal.ppat.1011796)
Supplement: S19 Fig — After 5 μM Ce6 or Ce6TME was sprayed on Nicotiana benthamiana for 7 days, the growth of tobacco was examined. This indicated that Ce6 or Ce6TME did not affect the growth and development of tobacco. (PDF) [file ppat.1011796.s019.pdf]

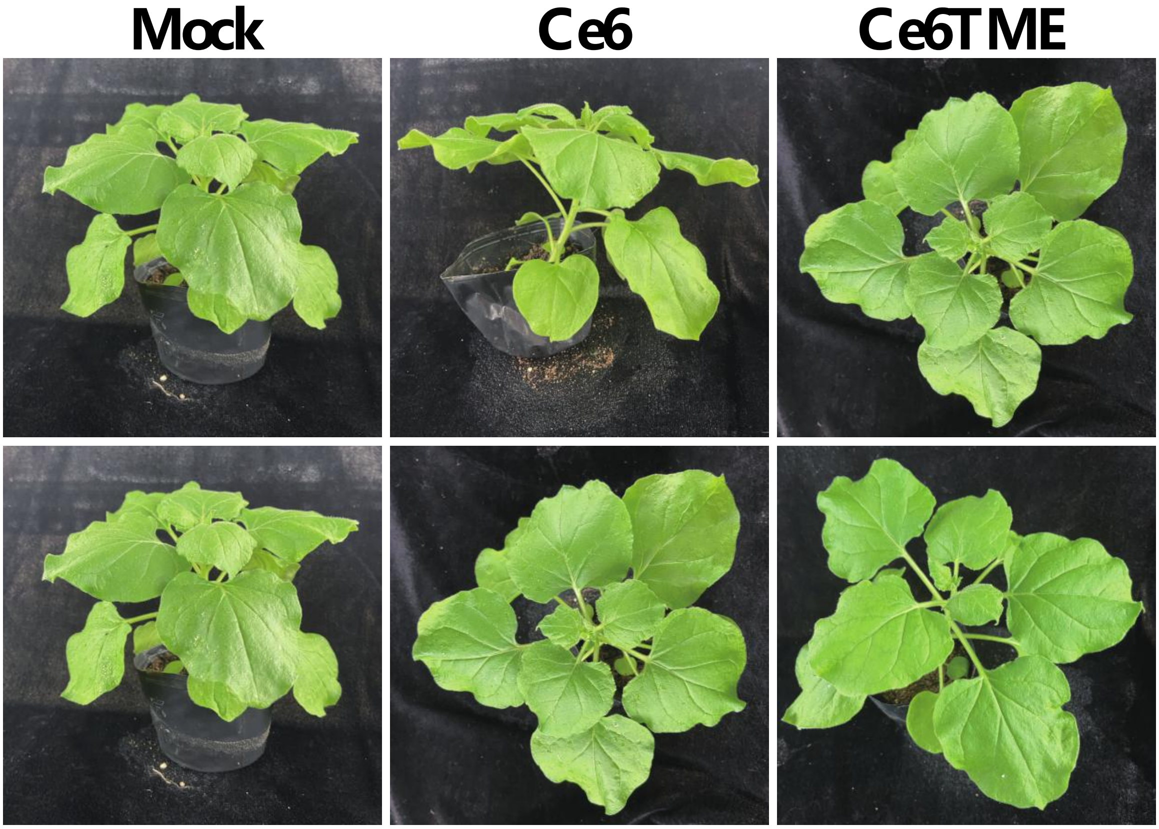

Fig S19. Phytotoxicity evaluation of chlorins on *Nicotiana benthamiana*. After 5 μM Ce6 or Ce6TME was sprayed on *Nicotiana benthamiana* for 7 days, the growth of tobacco was examined. This indicated that Ce6 or Ce6TME did not affect the growth and development of tobacco.
