# Supplementary material for: G-quadruplex in the TMV Genome Regulates Viral Proliferation and Acts as Antiviral Target of Photodynamic Therapy
Source: PLoS Pathog. 2023 Dec 7;19(12):e1011796. doi: 10.1371/journal.ppat.1011796 (PMC10760922; doi:10.1371/journal.ppat.1011796)
Supplement: S20 Fig — Fluorescent changes of resorufin were used to reflect the metabolic activities. (PDF) [file ppat.1011796.s020.pdf]

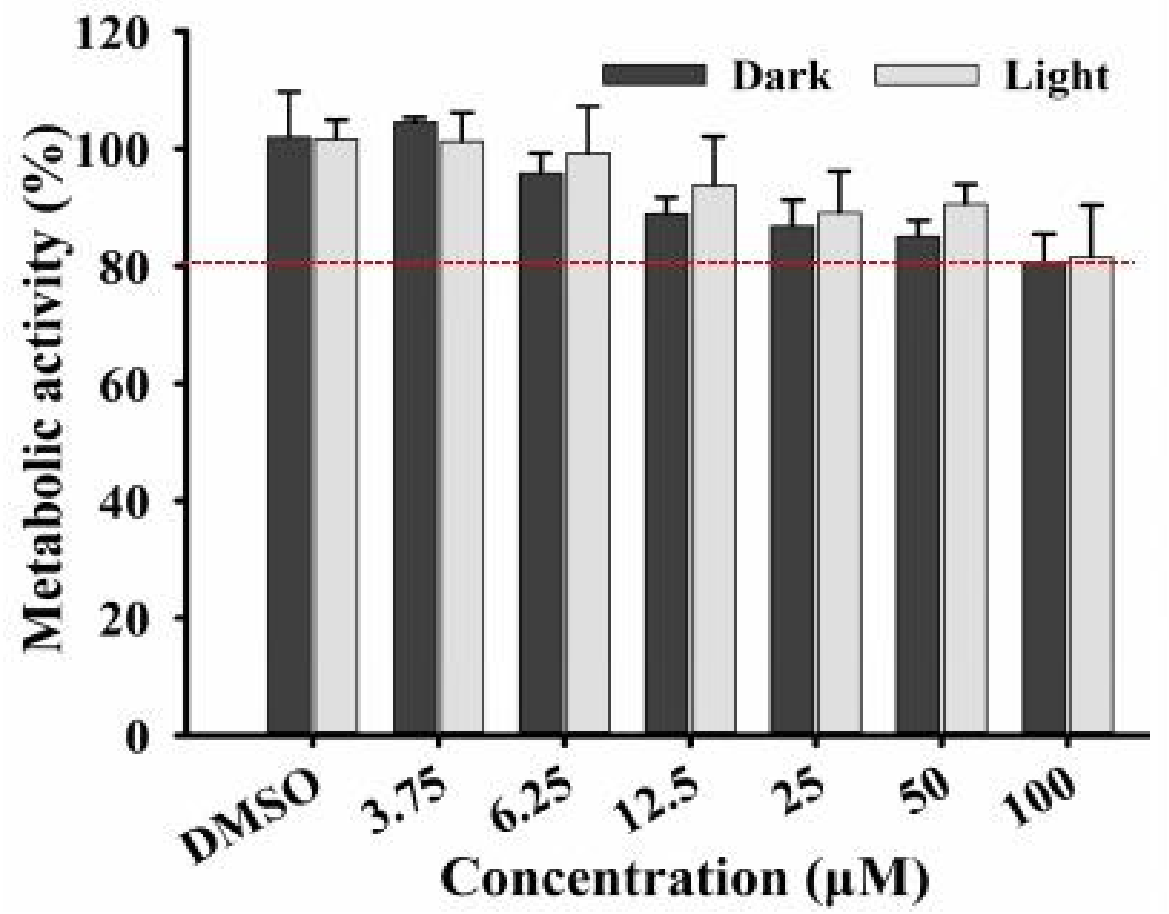

Fig S20. Metabolic activities of BY-2 cells treated with Ce6 at different concentrations under light or dark. Fluorescent changes of resorufin were used to reflect the metabolic activities.
