# Supplementary material for: G-quadruplex in the TMV Genome Regulates Viral Proliferation and Acts as Antiviral Target of Photodynamic Therapy
Source: PLoS Pathog. 2023 Dec 7;19(12):e1011796. doi: 10.1371/journal.ppat.1011796 (PMC10760922; doi:10.1371/journal.ppat.1011796)
Supplement: S1 Table — (PDF) [file ppat.1011796.s021.pdf]

Table S1. Reference genome sequences of plant viruses from 35 classified families

Genome type	ssDNA(493)	dsRNA(167)	ssRNA(+)(959)	ssRNA(-)(80)	dsDNA-RT(90)
Virus Family	Geminiviridae(389)	Amalgaviridae(10)	Alphaflexiviridae(57)	Aspiviridae(4)	Caulimoviridae(90)
	Genomoviridae(6)	Reoviridae(105)	Benyviridae(5)	Fimoviridae(4)	
	Nanoviridae(68)	Partitiviridae(33)	Betaflexiviridae(107)	Phenuiviridae(10)	
	Alphasatellitidae(5)	Totiviridae(19)	Bromoviridae(9)	Rhabdoviridae(26)	
	Bacilladnaviridae(5)		Closteroviridae(61)	Tospoviridae(36)	
	Circoviridae(6)		Luteoviridae(52)		
	Tolecusatellitidae(14)		Alvernaviridae(1)		
			Bromoviridae(117)		
			Endornaviridae(21)		
			Kitaviridae(15)		
			Marnaviridae(16)		
			Potyviridae(167)		
			Secoviridae(118)		
			Solemoviridae(20)		
			Togaviridae(2)		
			Tombusviridae(74)		
			Tymoviridae(38)		
			Virgaviridae(79)		
