# Supplementary material for: G-quadruplex in the TMV Genome Regulates Viral Proliferation and Acts as Antiviral Target of Photodynamic Therapy
Source: PLoS Pathog. 2023 Dec 7;19(12):e1011796. doi: 10.1371/journal.ppat.1011796 (PMC10760922; doi:10.1371/journal.ppat.1011796)
Supplement: S2 Table — (PDF) [file ppat.1011796.s022.pdf]

Table S2. Pattern conservation, the sequence conservation, and the folding potential of PQSs in TMV genomes

PQS	Start	End	PatCons	PhaCons	GerpS	G4HunterS	PQS
PQS1(+)	2561	2582	1	0.871	0.112	1	GGACGGAGUUCCGGGCUGUGG
PQS2(+)	3059	3073	0.808	0.79	0.071	1	GGAGAUGGUCGGCGG
PQS3(+)	4709	4728	0.731	0.819	0.141	0.95	GGAUUGGGAACACUUGGAGG
PQS4(+)	5113	5136	0.962	0.788	0.044	0.833	GGUUUGGUCGUCACGGGCGAGUGG
PQS5(+)	5178	5194	0.885	0.84	0.105	0.882	GGUGGACAAAAGGAUGG
PQS6(+)	6159	6178	1	0.872	0.192	0.55	GGUUUGGUUUGGACCUCUGG
PQS1(-)	2906	2927	0.885	0.813	-0.024	1.591	GGCAAAATGGGCGGGGTACGGG
PQS2(-)	6369	6383	0.885	0.863	0.21	2.867	GGGGGTAACGGGGGG

Note: To ensure confidence in the conservative analysis of G-quadruplex, all tobacco mosaic virus genome sequences in the NCBI database were collected, and classified according to the time and place of first discovery. Representative 26 virus strains in the same region and age were selected to ensure the diversity in the final data. Accession numbers of the twenty-six TMV genomes employed in the analysis were listed as follow: AF395127.1, AB369276.1, AB369275.1, JF920727.1, FR878069.1, HE818417.1, HE818457.1, HE818413.1, HE818455.1, KF972434.1, KF972431.1, KF972428.1, KF280646.1, MG516107.1, KY810785.1, MH595921.1, MH595920.1, MH595919.1, MN912489.1, OK149218.1, MT737799.1, MZ357187.1.

"PQS" represents the Potential G-quadruplex Sequence in the genome. "Start" represents the start site, and "End" represents the end sites of PQS in the TMV genome. "PatternCons" is the mode conservation, which is the number of viruses containing PQS at the same position divided by the total number of viruses [5]. "PhastCons" and "Gerpscore" represent sequence conservancy values [6, 7]. G4HunterScore is employed to evaluate the probability that PQS can fold into a G-quadruplex structure [8].
