# Supplementary material for: G-quadruplex in the TMV Genome Regulates Viral Proliferation and Acts as Antiviral Target of Photodynamic Therapy
Source: PLoS Pathog. 2023 Dec 7;19(12):e1011796. doi: 10.1371/journal.ppat.1011796 (PMC10760922; doi:10.1371/journal.ppat.1011796)
Supplement: S3 Table — (PDF) [file ppat.1011796.s023.pdf]

Table S3. Sequences of oligomers used in RNA stop assay

Oligomer	Sequence (5'-3')
Template TMV PQS5	GGUGGACAAAAGGAUGGAAUCGUAUAGUGAGUCGUAUUA
Template TMV PQS5-mut	GAUAGAACAAAAAGAUAAAAUCGUAUAGUGAGUCGUAUUA
RNA primer 15	FAM-UAAUACGACUCACUA
Marker 39	FAM-UAAUACGACUCACUAUACGAUCCCGCCCACCCGCAGCCC
Marker 21	FAM-UAAUACGACUCACUAUACGAU
Marker 17	FAM-UAAUACGACUCACUAUA

Note: the template TMV PQS5-mut was used in RNA stop assay.
