# Supplementary material for: G-quadruplex in the TMV Genome Regulates Viral Proliferation and Acts as Antiviral Target of Photodynamic Therapy
Source: PLoS Pathog. 2023 Dec 7;19(12):e1011796. doi: 10.1371/journal.ppat.1011796 (PMC10760922; doi:10.1371/journal.ppat.1011796)
Supplement: S4 Table — (PDF) [file ppat.1011796.s024.pdf]

Table S4. List of primers for reverse transcription and real time RT-PCR

Gene Name	Primer name	Primer squence(5'-3')	Usage
mRNA	oligo dT(18)	TTTTTTTTTTTTTTTTTT	RT-PCR
TMV coat protein	TMV-*cp*-RT	CACGTGTGATTACGGACACA	RT-PCR
	TMV-*cp*-F	TAGAGTAGACGACGCAACGG	QPCR
	TMV-*cp*-R	AGAGGTCCAAACCAAACCAG	
NbEF1α	NbEF1α-F	CCTTCTTGAGGCTCTTGACC	QPCR
	NbEF1α-R	GACACCAGTTTCCACACGAC	
