# Supplementary material for: G-quadruplex in the TMV Genome Regulates Viral Proliferation and Acts as Antiviral Target of Photodynamic Therapy
Source: PLoS Pathog. 2023 Dec 7;19(12):e1011796. doi: 10.1371/journal.ppat.1011796 (PMC10760922; doi:10.1371/journal.ppat.1011796)
Supplement: S5 Table — (PDF) [file ppat.1011796.s025.pdf]

Table S5. Antiviral activities of NMM against TMV in vivo

Compounds	Concentration (mg/L)	Inactivating Activity (%)	Curative Activity (%)	Protection Activity (%)
NMM	500	43.1±3.4	49.8±4.5	38.9±4.1
	100	19.5±1.7	5.2±1.0	9.1±0.9
Ningnanmycin	500	55.9±2.6	54.7±1.7	57.5±3.0
	100	25.3±2.0	26.9±1.4	28.3±0.6
Ribavirin	500	38.1±1.2	37.6±0.8	39.4±1.8
	100	11.5±0.7	14±2.3	12.6±0.5

Note: The values examined in inactivating activity of ningnanmycin is a little different with that in Table1 because of different testing institutions, but the differences are tolerable.
