# Supplementary material for: G-quadruplex in the TMV Genome Regulates Viral Proliferation and Acts as Antiviral Target of Photodynamic Therapy
Source: PLoS Pathog. 2023 Dec 7;19(12):e1011796. doi: 10.1371/journal.ppat.1011796 (PMC10760922; doi:10.1371/journal.ppat.1011796)
Supplement: S6 Table — (PDF) [file ppat.1011796.s026.pdf]

Table S6. Information of primers used in plasmid construction

Primers	Sequence (from 5' to 3')
TMM015	CAGATTAGTGACTGAGTACAAGGCCTCTGTGGACATGCC TGCGC
TMM016	CAACGACTTCTTCTGTAAGTTCCATGGGCCCTCCGTCTCT CACGTT
TMM021	GTGTCTTGTTGACAAAAGGATGG
TMM022	CCATCCTTTTGTCAACAAGACAC
TMM029	GTGTGTCTAGTAGACAAAAGGATGG
TMM030	CCATCCTTTTGTCTACTAGACACAC
TMM031	TGTGTCTAGTGGACAAAAGAATGG
TMM032	CCATTCTTTTGTCCACTAGACACA
TMM076	CGCGGGATATCATCGACCCGACGCCCAGGCCTCTGTGGA CATGCCT
TMM077	GCCGCACTAGTTCCAGGGCGCCCGGGCCCTCCGTCTCTC AC
TMM080	TCTGATCCAAGCTCAAGCTAAGCTTGAGCTCTCCCATAT GGTCGACT
TMM081	AGGTGAACCTTCTTAAAGGAGTTAAGCTTATTGATAGTG GATACGTCTGTTTA
TMM082	ATAACCACCCAGGACGCGAT
TMM083	ATCGCGTCCTGGGTGGTTAT
