# Supplementary material for: G-quadruplex in the TMV Genome Regulates Viral Proliferation and Acts as Antiviral Target of Photodynamic Therapy
Source: PLoS Pathog. 2023 Dec 7;19(12):e1011796. doi: 10.1371/journal.ppat.1011796 (PMC10760922; doi:10.1371/journal.ppat.1011796)
Supplement: S7 Table — (PDF) [file ppat.1011796.s027.pdf]

Table S7. Anti-TMV activities of different photosensitive compounds *in vivo*

Compounds.	Conc. (μg/mL)	Inactivating activity (%)	Protective activity (%)	Curative activity (%)
PP-a	100	12.8 ± 7.6	11.9 ± 8.7	--
	500	33.4 ± 9.6	22.3 ± 3.4	--
HD	100	29.5 ± 5.0	14.8 ± 4.4	13.6 ± 5.9
	500	49.4 ± 2.3	28.9 ± 6.9	38.8 ± 8.2
MPCl	100	--	5.5 ± 7.8	--
	500	19.1 ± 3.1	12.9 ± 1.0	11.7 ± 7.3
Ce6	100	37.0 ± 7.8	23.5 ± 6.0	23.0 ± 10.1
	500	85.4 ± 7.4	69.9 ± 2.5	63.8 ± 3.0
Ce6T	100	16.9 ± 6.6	19.1 ± 5.9	19.0 ± 6.6
	500	61.1 ± 5.0	52.7 ± 6.3	48.2 ± 1.6
Ce6TME	100	9.1 ± 3.1	--	1.1 ± 1.3
	500	26.9 ± 6.4	20.3 ± 5.2	31.3 ± 1.1
Ningnanmycin	100	40.8 ± 5.0	27.3 ± 0.3	30.1 ± 9.6
	500	84.9 ± 1.9	59.1 ± 1.6	65.4 ± 4.6
Ribavirin	100	11.2 ± 2.5	10.7 ± 5.1	10.4 ± 1.9
	500	42.2 ± 8.3	36.6 ± 4.9	33.2 ± 0.7

Note. Ningnanmycin and Ribavirin were used as positive controls in this experiment.
